# SREKA-targeted liposomes for highly metastatic breast cancer therapy

**DOI:** 10.1080/10717544.2023.2174210

**Published:** 2023-02-08

**Authors:** Balázs Vári, Levente Dókus, Adina Borbély, Anikó Gaál, Diána Vári-Mező, Ivan Ranđelović, Anna Sólyom-Tisza, Zoltán Varga, Norbert Szoboszlai, Gábor Mező, József Tóvári

**Affiliations:** a National Institute of Oncology, Department of Experimental Pharmacology, National Tumor Biology Laboratory, Budapest, Hungary; b School of Ph.D. Studies, Semmelweis University, Budapest, Hungary; c Research Group of Peptide Chemistry, Eötvös Loránd Research Network, Budapest, Hungary; d Faculty of Science, Institute of Chemistry, Eötvös Loránd University, Budapest, Hungary; e MTA-ELTE Lendület Ion Mobility Mass Spectrometry Research Group, Budapest, Hungary; f Eötvös, Loránd Research Network, Research Centre for Natural Sciences, Institute of Materials and Environmental Chemistry, Biological Nanochemistry Research Group, Budapest, Hungary; g Department of Tumor Biology, National Korányi Institute of Pulmonology, Budapest, Hungary

**Keywords:** Targeted cancer therapy, nanocarriers, SREKA peptide, tumor metastasis

## Abstract

Chemotherapy is still a leading therapeutic approach in various tumor types that is often accompanied by a poor prognosis because of metastases. PEGylated liposomes with CREKA targeting moiety are well-known therapeutic agents, especially in highly metastatic experimental models. CREKA specifically targets tumor-associated ECM, which is present at the primary, as well as metastatic tumor sites. To better understand the function of the targeting moieties, we decided to design various liposome formulations with different amounts of targeting moiety attached to their DSPE-PEG molecules. Moreover, a new tumor-homing pentapeptide (SREKA) was designed, and a novel conjugation strategy between SREKA and DSPE-PEGs. First, the *in vitro* proliferation inhibition of drug-loaded liposomes and the cellular uptake of their cargo were investigated. Afterward, liposome stability in murine blood and drug accumulation in different tissues were measured. Furthermore, *in vivo* tumor growth, and metastasis inhibition potencies of the different liposome formulations were examined. According to our comparative studies, SREKA-liposomes have a uniform phenotype after formulation and have similar characteristics and tumor-homing capabilities to CREKA-liposomes. However, the exchange of the N-terminal cysteine to serine during conjugation results in a higher production yield and better stability upon conjugation to DSPE-PEGs. We also showed that SREKA-liposomes have significant inhibition on primary tumor growth and metastasis incidence; furthermore, increase the survival rate of tumor-bearing mice. Besides, we provide evidence that the amount of targeting moiety attached to DSPE-PEGs is largely responsible for the stability of liposomes, therefore it plays an important role in toxicity and targeting.

## Introduction

**Figure 1.**
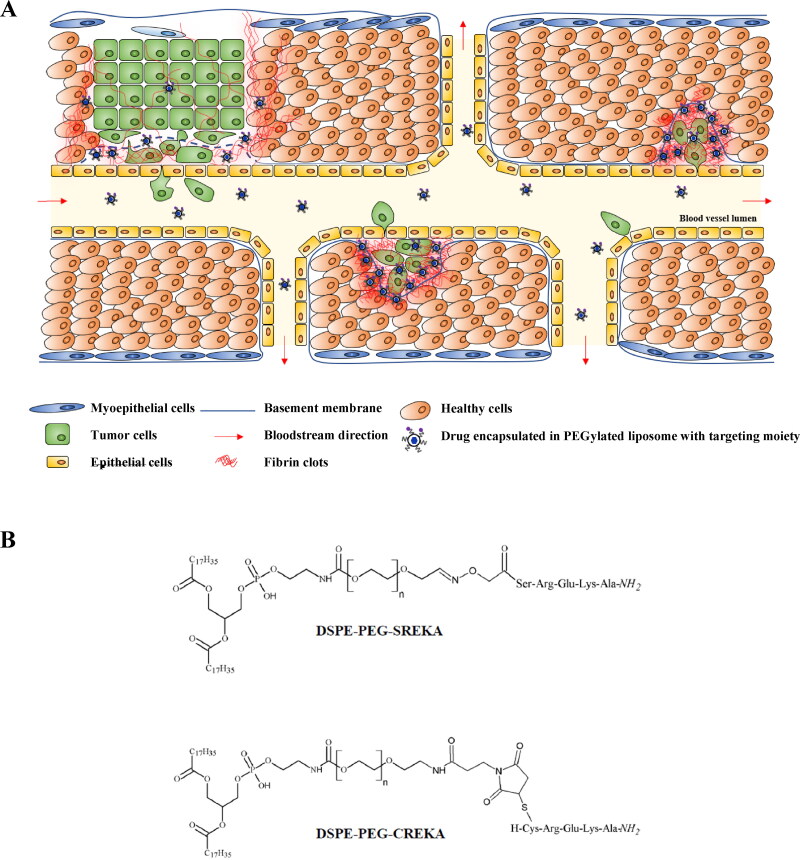
The process of metastasis and the working principle of newly developed targeted liposomes. A: Metastatic breast cancer can develop when the basement membrane is broken down, and it allows malignant cells to migrate from their primary site into the bloodstream or lymphatic vessels (intravasation). When circulating tumor cells adhere to distant locations inside the vessel, extravasation occurs, and cells invade the distant tissue. If these tumor cells can survive in their new microenvironment and start to proliferate, metastatic sites are established. Cancer cells and tumor-associated fibroblasts produce an increased amount of fibrin, resulting in the accumulation of fibrin clots in proximity to the primary tumor and especially at metastases. PEGylated liposomes are stable drug carriers, which, once injected into patients, stay inside the bloodstream for an extended amount of time compared to the free drug. Modification of PEG molecules with SREKA enables liposomes to specifically bind to fibrin clots associated with primary and metastatic tumor sites resulting in the accumulation of liposomes at these sites and the release of their cargo. B: DSPE-PEG2000 conjugate of Aoa-SREKA-NH2 peptide derivative with oxime-bond (upper) and DSPE-PEG2000 conjugate of H-CREKA-NH2 peptide with thioether bond (lower).

Cancer is a leading cause of death worldwide and a significant barrier to increasing lifespan in most developed countries. According to estimations, in 2020, there were over 19 million new cancer cases worldwide. Moreover, around 10 million deaths were caused by malignant diseases, and these numbers seem to increase year by year. The most common cancer type in 2020 was breast cancer, which was responsible for over 2.2 million deaths. Even though the 5-year survival rate of localized breast cancer is relatively high, the presence of metastatic sites in distant organs decreases survival rates drastically (Weigelt et al., 2005). Thus, inhibiting the development of metastatic events is crucial to increasing the standard of care for patients suffering from breast cancer. Aside from surgical removal of tumors, traditional chemotherapy is still one of the primary modalities to treat cancer. However, cytotoxic anticancer agents may cause several serious side effects due to the lack of selectivity. To overcome these drawbacks, researchers are focused on the enhancement of the specificity of drugs toward tumor cells, including the application of targeted drugs or nanoparticles filled with cytotoxic agents (Kumari et al., 2016; Vrettos et al., 2018). Nanocarriers such as liposomes (e.g. Doxil®, the first FDA-approved nano-drug for the treatment of AIDS-related Kaposi’s sarcoma, breast cancer, ovarian cancer, and other solid tumors (Barenholz, 2012)) have been successfully used in tumor therapy because of their prolonged half-life in the bloodstream, and enhanced accumulation of drugs in tumor tissue (Allen & Cullis, 2013). Nevertheless, it is hypothesized that the drug selectivity can be further increased by the attachment of homing devices (e.g. tumor-specific peptides) to the surface of nanocarriers, especially in cases of the treatment of metastatic cancers (Aronson et al., 2021). To inhibit tumor cell migration and invasion, Cys-Arg-Glu-Lys-Ala (CREKA) homing peptide was recently investigated to deliver PEGylated liposomes filled with doxorubicin (Jiang et al., 2018). CREKA pentapeptide was selected by *in vivo* phage display, which selectively targets tumor blood vessels (Hoffman et al., 2004; Simberg et al., 2007). It was also indicated that this homing motif has a specific affinity toward deposited fibrin-fibronectin clots, which accumulate in tumor ECM but not in healthy tissues, and are involved in the metastatic processes (Pilch et al., 2006). Therefore, the CREKA homing peptide is suitable for the delivery of cargo molecules to tumors by binding to neoangiogenic vessels and tumor stroma (***Figure 1***(A)).

During the development of CREKA-decorated nanoparticles, in most cases, the peptide was attached to PEGylated phospholipid (DSPE-PEG) derivatives through a maleimide functional group by thiol-ene click reaction (Jiang et al., 2018; Pilch et al., 2006; Wang et al., 2015). In this reaction, a thioether bond was formed from the thiol group of Cys, meaning that the free thiol group is not essential for receptor recognition. It is worth mentioning that the unprotected N-terminal Cys is extremely sensitive to oxidation; therefore, the dimerization of the peptide competes with the ligation procedure. This fact makes it necessary to apply oxygen-free conditions to avoid the formation of a dimer as a side product (Mezö et al., 2004). Furthermore, the positively charged N-terminal amino group may increase the nonspecific internalization rate and decrease blood circulation half-life (de Jesús Ruíz-Baltazar et al., 2017).

However, there are many contradictions in the literature regarding the structure of the C-terminal site of the CREKA peptide. In general, there is no information about the composition of the peptide bought from companies. On the other hand, when data is available, both a free carboxyl group and its amidated versions are mentioned. When synthesis steps are presented, Rink-Amide resin is used for solid phase synthesis that allows the presence of carboxamide C-terminal after the cleavage of the peptide from resin (Chung et al., 2014). Moreover, several groups prepared longer peptides using the CREKA motif at the N-terminus, suggesting that the free carboxyl group is not crucial for its biological activity (Soler et al., 2014; Wan et al., 2019). In addition, the peptide in the phage display technique is connected through its C-terminus to phages; therefore, they do not contain the free COOH group when tested.

In this study, the efficiency of a newly developed pentapeptide, Ser-Arg-Glu-Lys-Ala (SREKA) as homing peptide was investigated and studied in comparison with the amidated CREKA derivative. Using different chemoselective ligation procedures, the SREKA peptide was attached to the functionalized PEGylated phospholipids through its N-terminal amino group. This way, the positive charge was eliminated from the N-terminus of the homing moiety, and the drawbacks that apply to CREKA might be eliminated. The efficacy of the synthesis and ligation procedures were compared with CREKA-liposomes. Cellular uptake and proliferation inhibition of SREKA- and CREKA liposomes, as well as the free drug were tested in vitro. In addition, primary tumor growth and metastasis inhibition were investigated in a highly metastatic 4T1 epithelial murine breast cancer-bearing mouse model.

Moreover, we aimed to better understand the nature of targeting moieties attached to liposomes. Therefore, we investigated the effect of the modification of DSPE-PEG derivatives used for liposome preparation by conjugating different amounts of targeting moiety upon liposome production. Besides tumor inhibition abilities, stability of liposomes in murine blood, and uptake of their cargo by heart, lung, kidney, liver, spleen, and primary tumor were analyzed. Finally, we examined the effect of liposomes on median survival.

## Materials and methods

### Materials and animals

Solvents for the syntheses and purification were obtained from Reanal (Budapest, Hungary) or VWR International Kft. (Debrecen, Hungary). All amino acid derivatives used for the synthesis of peptides and Fmoc-Rink-amide MBHA resin were purchased from Iris Biotech GmbH (Marktredwitz, Germany). Reagents applied for SPPS [*N*,*N*′-diisopropylcarbodiimide (DIC), 1,8-diazabicyclo[5.4.0]undec-7-ene (DBU), 1-hydroxybenzotriazole hydrate (HOBt), triisopropylsilane (TIS)], ninhydrin, acetic anhydride, *N*,*N*-diisopropylethylamine were delivered by Sigma-Aldrich, St. Louis, Missouri, USA. Aminooxyacetic acid and methoxyamine were TCI (Tokyo, Japan) products. Daunomycin was donated from IVAX (Budapest Hungary). The PEGylated phospholipid derivatives were purchased from Biopharma PEG (Watertown, USA).

Synthetic high-purity hydrogenated soybean phosphatidylcholine (HSPC), 3β-hydroxy-5-cholestene (cholesterol), 1,2-distearoyl-sn-glycero-3-phosphoethanolamine-*N*-[amino(polyethylene glycol)_2000_] (ammonium salt) (DSPE-PEG_2000_), and 1,2-distearoyl-*sn*-glycero-3-phosphoethanolamine-*N*-[biotinyl(polyethylene glycol)_2000_] (ammonium salt) (DSPE-PEG_2000_) were obtained from Avanti Polar Lipids (Alabaster, AL, USA) or Merck Life Science Kft. (Merck KGaA, Darmstadt, Germany). The DSPE-PEG_2000_-SREKA and DSPE-PEG_2000_-CREKA were synthesized as described later. All chemicals: chloroform (LiChrosolv®, Sigma-Aldrich), hydrochloric acid (1 M, Reag. Ph Eur, Reag. USP, Titripur®, Supelco), formic acid (LC-MS grade, Fischer Chemical, Thermo Fisher Scientific), sodium hydroxide (1 M, Reag. Ph Eur, Reag. USP, Titripur®, Supelco), l-histidine ((*S*)-2-amino-3-(4-imidazolyl)propionic acid, BioUltra, ≥99.5%, Sigma-Aldrich), d-(+) saccharose (puriss, PH. EUR, Sigma-Aldrich), ammonium sulfate (Molecular Biology Tested, ≥99%, Sigma-Aldrich) was purchased from Merck Life Science Kft (Budapest, Hungary). Each buffer was filtered through 0.22-µm filters (Steritop Vacuum Driven Disposable Filtration System, Merck KGaA or Acrodisc Syringe Filter, PALL Life Sciences, Pall Corporation, New York, USA). HPLC-MS grade acetonitrile, methanol, and water were purchased from VWR International Kft. (Debrecen, Hungary). Deionized Milli-Q (Millipore, Molsheim, France) water with a resistivity of 18.2 MΩ·cm was used throughout the experiments.

Adult female inbred Balb/c mice were bred in a specified pathogen-free (SPF) environment in the National Institute of Oncology (Budapest, Hungary). Mice were used in chronic toxicity studies and in orthotopic 4T1 murine breast tumor model experiments. Mice were kept in a sterile environment in Makrolon® cages at 22–24 °C (40–50% humidity), with light regulation of 12/12 h light/dark. The animals had free access to tap water and were fed a sterilized standard diet (VRF1, autoclavable, Akronom Kft., Budapest, Hungary) ad libitum. Animals used in our study were taken care of according to the “Guiding Principles for the Care and Use of Animals” based on the Helsinki declaration, and they were approved by the ethical committee of the National Institute of Oncology. Animal housing density was according to the regulations and recommendations from directive 2010/63/EU of the European Parliament and of the Council of the European Union on the protection of animals used for scientific purposes. Permission license for breeding and performing experiments with laboratory animals: PEI/001/1738-3/2015 and PE/EA/1461-7/2020.

### Synthesis of peptide derivatives

Peptide derivatives were synthesized manually by solid-phase peptide synthesis (SPPS), using the standard protocol of the Fmoc/*^t^*Bu strategy. For the synthesis 0.3 g Fmoc-Rink-amide MBHA resin (0.64 mmol/g capacity) was used. The Fmoc group was cleaved by 2% DBU and 2% piperidine in DMF solution four times (2, 2, 5, and 10 min, respectively), followed by couplings of the amino acid derivatives with DIC-HOBt coupling agents (3 equiv. each to the resin capacity) in DMF for 60 min.

For the preparation of aminooxyacetyl functionalized homing peptide, isopropylidene-protected derivative of aminooxyacetic acid was attached to the N-terminus using DIC and HOBt, similarly to the coupling of common amino acid derivatives.

The protecting groups were cleaved, and the peptides were removed from the resin by using 5 mL of TFA, 125 µL of distilled water, and 125 µL of TIS cleavage mixture at RT for 3 h. The crude products were precipitated by dry diethyl ether, dissolved in 10% acetic acid, freeze-dried, and purified by RP-HPLC (Gradient I).

### Synthesis of PEGylated phospholipid conjugates

#### Development of oxime linkage between the aminooxyacetyl-peptide and aldehyde-functionalized DSPE-PEG analog

In the first step, the isopropylidene-protecting group was cleaved from the peptide derivative with 1.5 M methoxyamine in 0.2 M NH_4_OAc buffer (pH = 5) at RT for 2 h. The unprotected product was isolated by RP-HPLC (Gradient II) and freeze-dried.

The conjugation between the freeze-dried peptide and phospholipid derivative was achieved in a mixture solvent (40% NH_4_OAc buffer (0.2 M, pH = 5); 40% Cellosolve (2-ethoxy-ethanol) and 20% acetonitrile (vol/vol)) in an overnight reaction (Supplementary Scheme S2A). The aminooxyacetyl-peptide was applied (2 equiv.) to the fatty acid derivative which was dissolved at 20 mg/mL concentration. The separation of the reaction mixture was carried out by RP-HPLC (Gradient III), and the identified fractions (by MS) of the conjugate were freeze-dried.

#### Conjugation of the CREKA homing moiety with the maleimide functionalized fatty acid derivative

The free thiol group of H-CREKA-NH_2_ peptide was used to form a thioether bond with the maleimide moiety of a functionalized fatty acid derivative (Supplementary Scheme S2B). Twenty mg of DSPE-PEG_2000_-Mal was dissolved in N_2_-purged PBS buffer (pH = 7), and the peptide compound (2 equiv.) was added into the solution in 10 equal portions under one hour. After a 20 h reaction time at RT, the compounds were separated by RP-HPLC. The fractions with conjugates were lyophilized before the MS characterization.

### Reverse phase high-performance liquid chromatography

The purification of the crude peptide derivatives was carried out by reverse phase high-performance liquid chromatography (RP-HPLC) technique using KNAUER 2501 HPLC system (Bad Homburg, Germany) and Phenomenex Luna (Torrance, CA, USA) C18 column (250 × 21.2 mm I.D.) with 10 µm silica (100 Å pore size). Experiments were carried out at a flow rate of 14 mL/min at RT, in the cases of Gradients I and II.

The isolation of the final conjugates from the reaction mixtures was carried out also by RP-HPLC method using KNAUER 2501 HPLC system and Phenomenex Jupiter C4 column (250 × 10 mm I.D.) with 10 µm silica (300 Å pore size), with 4 mL/min flow rate at RT (Gradient III). Eluent A was 0.1% TFA in distilled water and eluent B was 0.1% TFA in CH_3_CN-water (80:20, vol/vol). Peaks were detected at *λ* = 220 nm where the peptide-modified DSPE-PEG conjugates provide appropriate absorbance for detection. In all cases, linear gradient elution was applied. The gradients were as follows: Gradient I: 0 min 20% B, 5 min 20% B, 50 min 80% B; Gradient II: 0 min 5% B, 12 min 5% B, 12.1 min 100% B 50 min 100% B; Gradient III: 0 min 10% B, 10 min 10% B, 10.1 min 20% B, 50 min 60% B.

Analytical RP-HPLC was performed on an Exformma 1600 system using Waters Symmetry (WAT 045905) C18 column (150 × 4.6 mm I.D.) with 5 µm silica (100 Å pore size) as a stationary phase. The same eluents were applied as described earlier. A linear gradient elution was developed: 0 min 0% B; 2 min 0% B; 22 min 90% B with eluent. A flow rate of 1 mL/min was used at ambient temperature. Samples were dissolved in eluent A, and 20 μL was injected. Peaks were detected at *λ* = 220 nm.

### MS and HPLC-MS analysis for compound identification

The identification of the peptide derivatives and their DSPE-PEG conjugates was achieved by electrospray ionization mass spectrometry (ESI-MS) on a Bruker Daltonics Esquire 3000 Plus (Bremen, Germany) ion trap mass spectrometer, operating in continuous sample injection with 4 µL/min flow rate. Samples were dissolved in CH_3_CN-water mixture (50:50 vol/vol%) containing 0.1 vol/vol% AcOH. Mass spectra were recorded in positive ion mode in the *m*/*z* 50–2000 range.

Before the biological characterization, the purity of the conjugate as the final product was investigated on a Q ExactiveTM Focus, high resolution and high mass accuracy, hybrid quadrupole-orbitrap mass spectrometer (Thermo Fisher Scientific, Bremen, Germany) using an online UHPLC coupling. UHPLC separation was performed on a Dionex 3000 UHPLC system using a Supelco Ascentis C18 column (2.1 × 150 mm, 3 µm). Linear gradient elution (0 min 2% B, 1 min 2% B, 17 min 90% B) with eluent A (0.1% HCOOH in water, vol/vol) and eluent B (0.1% HCOOH in acetonitrile/water, 80:20, vol/vol) was used at a flow rate of 0.2 mL/min at 40 °C. High-resolution mass spectra were acquired in the 200–1600 *m*/*z* range. LC-MS data were analyzed by XcaliburTM software (Thermo Fisher Scientific) and with Origin Pro 8 (OriginLab Corp., Northampton, MA, USA).

### Preparation of CREKA/SREKA modified liposomes

CREKA/SREKA peptide-containing liposomes (***Table 1***) were prepared by the lipid film hydration and extrusion method. Stock solutions of DSPE-PEG-CREKA (5 mg/mL) and DSPE-PEG-SREKA (10 mg/mL) in acetonitrile were added to the lipid mixture containing HSPC, cholesterol, DSPE-PEG_2000_ dissolved in chloroform and dried to a thin lipid film under a stream of N_2_ gas, followed by incubation overnight under vacuum to remove residual solvent. Next, 0.25 M, pH = 6.5 ammonium sulfate solution was used to hydrate the lipid films to gain a total lipid concentration of 16 mg/mL (9.6 mg/mL HSPC, 3.2 mg/mL cholesterol, and 3.2 mg/mL DSPE-PEG_2000_ or the peptide modified PEG lipids (Table 1). Then the mixture was kept in 60 °C for 30 min using a magnetic hot plate (IKA RET control-visc, IKA-Werke GmbH & Co. KG, Staufen, Germany). The resulting multilamellar vesicle (MLV) suspension was subjected to five cycles of freeze-and-thaw (5 min each, freezing in liquid nitrogen, and thawing at 60 °C) before being extruded 10 times at 60 °C through a 100 nm polycarbonate membrane filter (Whatman, Springfield Mill, UK). The buffer was changed to l-Histidine/saccharose buffer (10 mM/10%, pH = 6.5) using a PD-10 column (Sephadex G-25, Cytiva, Little Chalfont, England). Next, daunomycin (7 mg/mL in 0.9% NaCl) was added to the liposomes (3.5 mL liposome sample + 1.5 mL 7 mg/mL daunomycin solution), followed by incubation for 1 h at 60 °C. The unencapsulated daunomycin drug was removed using PD-10 or G-25 midiTrap desalting columns according to the manufacturer’s instructions. A drug-free liposomal formulation was also prepared with a lipid composition corresponding to the Lipo-25S sample, hereinafter named E-Lipo-25S.

**Table 1.** Characteristics of liposome formulations.

	DSPE-PEG	CREKA-PEG	SREKA-PEG	Mean diameter (nm) ± SD (nm)	PDI (%)	Daunomycin concentration (mg/mL)
Lipo-NP	100%	0%	0%	120.54 ± 14.24	11.81	1.48
Lipo-25C	75%	25%	0%	112.37 ± 16.23	14.44	1.43
Lipo-50C	50%	50%	0%	124.21 ± 21.40	17.23	1.33
Lipo-100C	0%	100%	0%	126.88 ± 30.66	24.17	1.53
Lipo-25S	75%	0%	25%	117.93 ± 18.34	15.55	1.41
Lipo-50S	50%	0%	50%	135.63 ± 24.78	18.27	1.39
Lipo-100S	0%	0%	100%	131.11 ± 34.06	25.98	1.52
E-Lipo-25S	75%	0%	25%	139.68 ± 19.19	13.74	0

The composition of liposomes is shown in columns 2–4. Mean diameter and polydispersity indexes (PDIs) of the prepared liposomes measured by DLS method are shown in columns 5–6 and daunomycin content can be seen in column 7.

### Characterization of liposomes

The size distribution of the different liposomes was determined by dynamic light scattering (DLS) using a 10× dilution in 0.9% NaCl solution at 20 °C. DLS measurements were performed on a W130i apparatus (Avid Nano Ltd., High Wycombe, UK) and using a low-volume disposable cuvette (UVette, Eppendorf Austria GmbH, Vienna, Austria), which was equipped with a diode laser (*λ* = 660 nm) and a side scatter detector at a fixed angle of 90°. Data evaluation was performed with pUNk1003 software (Avid-Nano), utilizing the CONTIN algorithm.

Zeta potentials of the different liposomes were measured by Malvern Zetasizer Nano ZS (Malvern, Worcs, UK) equipped with He–Ne laser (*λ* = 633 nm) and backscatter detector at a fixed angle of 173°. The measurements were performed with liposome formulations 8× diluted by l-Histidine/saccharose buffer (10 mM/10%, pH = 6.5) at room temperature.

UV–vis spectrophotometry was used to determine the drug concentration of the prepared liposomes. Briefly, 100 µL of daunomycin-loaded liposomes were diluted with 1% HCl in absolute ethanol. The resultant solutions were measured using an EnSpire microplate reader (Perkin Elmer, Waltham, Massachusetts, USA) at the excitation wavelength of 498 nm and emission wavelength of 584 nm to determine the total amount of the daunomycin drug.

The entrapment efficiency (EE%), drug-to-lipid ratio, and drug loading (DL%) of the prepared liposomes were calculated according to the following formulae:

(1) $$\text{EE\% }=\frac{W\text{ entrapped drug}}{W\text{ initial drug}}\times 100$$

(2) $$\text{drug-to-lipid ratio}=\frac{\text{mol drug}}{\text{mol lipid}}$$

(3) $$\text{DL\% }=\frac{W\text{ entrapped drug}}{W\text{ total drug}+W\text{ of total lipid}}\times 100$$

where *W* represents the weight in mg.

The morphology of liposomes was observed by freeze-fracture combined transmission electron microscopy (FF-TEM). Liposome samples were mixed with glycerol (Sigma-Aldrich, St. Louis, Missouri, USA) which is used as a cryoprotectant at a 3:1 sample-to-glycerol volume ratio. Approximately 2 μL of the samples were pipetted onto a gold sample holder and frozen by placing it immediately into partially solidified Freon for 20 seconds. Fracturing was performed at –100 °C in a Balzers freeze-fracture device (Balzers BAF 400D, Balzers AG, Liechtenstein). The replicas of the fractured surfaces were made by platinum-carbon evaporation and then cleaned with a water solution of surfactant and washed with distilled water. The platinum-carbon replicas were placed on 200 mesh copper grids and examined in a MORGAGNI 268D (FEI, The Netherlands) transmission electron microscope.

### Cell culture and cellular uptake

4T1-Luc and NIH-3T3 cells were obtained from ATCC and were grown in RPMI1640 medium (BioSera, Nuaille, France) supplemented with 10% FBS (BioSera, Nuaille, France) and 1% penicillin/streptomycin (BioSera, Nuaille, France). Cells were maintained in sterile T75 flasks with ventilation caps (Sarstedt, Nümbrecht, Germany). Cells were trypsinized (BioSera, Nuaille, France) before reaching 95% confluency and reseeded with a subcultivation ratio of 1:6 to 1:8.

The cellular uptake studies were performed in 6-well plates (Sigma-Aldrich, St. Louis, Missouri, USA). Briefly, 10^5^ cells were seeded into each well. Forty-eight hours after seeding, 10 µg of daunomycin content was administered, and cells were incubated for 1, 4, and 24 h. Afterward, cells were washed two times with DPBS (Biosera, Nuaille, France), trypsinized, and collected into a 15-mL Falcon tube (LP Italiana, Milano, Italy). Cells were washed again two times with DPBS, then resuspended, and three freeze-thaw cycles were applied. About 600 µL of ice-cold acetonitrile (ACN):methanol (MeOH) = 9:1 mixture was added, the solution was vortexed thoroughly, and samples were centrifuged (Eppendorf centrifuge 5804 R) at 2500 × *g* for 15 min at 4 °C. The supernatant was collected and incubated overnight at –20 °C. Afterward, aqueous 0.1% formic acid was added at a 1:1 ratio to acidify and dilute the sample. Finally, HPLC-MS/MS analysis (see section HPLC-MS/MS analysis for cellular uptake, blood stability, and biodistribution studies) was performed to detect the level of daunomycin.

### *In vitro* proliferation inhibition assay

Cells were seeded into 96-well plates (Sigma-Aldrich, St. Louis, Missouri, USA). Twenty-four hours after seeding, cells were treated with the respective compound either for 24 h or for 72 h. In the case of 24-h treatment, the medium was exchanged for fresh growth medium after 24 h, and cells were incubated for another 48 h before proceeding. Treatment conditions were the following for all liposome formulations and for free daunomycin: 9-point dilution series starting from 100 µM with a DF = 4. After treatment, 0.5 mg/mL MTT (Duchefa, Haarlem, The Netherlands) was administered to each well, and the plate was incubated for 4 h. Afterward, the supernatant was removed, and 100 µL of DMSO:MeOH = 1:1 was added to wells. DMSO was obtained from Sigma-Aldrich, St. Louis, Missouri, USA. MTT crystals were resuspended thoroughly, and absorbance was measured at a wavelength of 570 nm with a microplate reader (CLARIOstarplus, BMG Labtech, Ortenberg, Germany). Three technical replicates were performed of the MTT. IC_50_ values were calculated using GraphPad Prism 6 software.

### Blood pharmacokinetics

The blood pharmacokinetics experiments were carried out in a murine orthotopic breast cancer allograft model. Briefly, 0.5 × 10^6^ of 4T1-Luc cells were inoculated in 0.1 mL RPMI1640 basic medium into the mammary fat pad of 8- to 12-week-old female Balb/c mice. Then, mice were treated with an equal amount of daunomycin (3 mg/kg), either encapsulated by liposomes or in free form. 1, 4, 24, 48, and 96 h after treatment 50 µL of blood was drawn from the orbital sinus following anesthesia with 2-Methylbutane ReagentPlus®, ≥99% (Sigma-Aldrich, St. Louis, Missouri, USA). Blood samples were frozen at –80 °C in Protein LoBind® tubes (Eppendorf, Hamburg, Germany) containing heparin (TEVA, Debrecen, Hungary). After all the samples were collected, 10 µL of 5% (wt/vol) ZnSO_4_ (ThermoFisher Scientific, Waltham, Massachusetts, USA) was added, and the mixture was vortexed thoroughly. Next, 150 µL of ice-cooled ACN:MeOH = 9:1 mixture was added, and the solution was vortexed and stored at –20 °C for 16 h to allow protein precipitation. Samples were centrifuged at 5000 × *g* for 30 min at 4 °C. The supernatant was removed, and aqueous 0.1% formic acid was added at a 1:1 ratio to acidify and dilute the sample. HPLC-MS/MS analysis (see section HPLC-MS/MS analysis for cellular uptake, blood stability, and biodistribution studies) was performed to detect the level of daunomycin.

### Biodistribution studies

Biodistribution studies were carried out in our murine orthotopic breast cancer allograft model. Tumor-bearing mice were treated with an equal amount of daunomycin (3 mg/kg) intraperitoneally, either encapsulated by liposomes or in a free form. Ninety-six hours after treatment, mice were euthanized using isoflurane (Baxter, Budapest, Hungary) at a concentration of 5% as suggested in the drug’s guidelines. Isoflurane exposure was continued until one minute after breathing stopped. Primary tumor, lung, heart, liver, spleen, and kidney were removed and snap-frozen in isopentane (Sigma-Aldrich, St. Louis, Missouri, USA). Organs and primary tumors were pooled based on treatment groups. Tissue samples were weighed accurately and homogenized in sterile distilled water (30%, wt/vol) with gentleMACS™ Dissociator (Miltenyi Biotec, Bergisch Gladbach, Germany). Homogenates were centrifuged at 500 × *g* for 5 min at RT. After gentle resuspension with a pipette, 200 µL was transferred into LoBind® tubes, and the leftover was frozen for further analysis. The precipitation of the proteins and the extraction of daunomycin from the different tissue homogenates were performed as described earlier. HPLC-MS/MS analysis (see section HPLC-MS/MS analysis for cellular uptake, blood stability, and biodistribution studies) was performed to detect the level of daunomycin.

### HPLC-MS/MS analysis for cellular uptake, blood stability, and biodistribution studies

Samples were analyzed by HPLC-MS/MS using a Dionex UltiMate 3000 system coupled with a Q Exactive^TM^ Focus, high resolution and high mass accuracy, hybrid quadrupole-orbitrap mass spectrometer (Thermo Fisher Scientific, Bremen, Germany) using a Supelco Ascentis C18 column (2.1 × 150 mm, 3 µm). Linear gradient elution (0 min 2% B, 1 min 2% B, 11 min 90% B, 11.5 min 90% B, 12 min 2% B, 15 min 2% B) with eluent A (0.1% HCOOH in water, vol/vol%) and eluent B (0.1% HCOOH in acetonitrile/water, 80:20, vol/vol%) was used at a flow rate of 0.2 mL/min at 40 °C. Detection of daunomycin was in ESI + mode using Parallel Reaction Monitoring (PRM) at a resolution of 17,500 FWHM. The precursor ion (*m/z*: 528.19) was selected for analysis. The isolation window with was set to 2 *m/z*. Normalized collision energy (NCE) was 15%. LC-MS/MS data were visualized and analyzed by Xcalibur^TM^ software (Thermo Fisher Scientific). Peak area from the Extracted Ion Chromatograms (EIC) of the ion transition *m/z* 528.19 -> 321.07 (±0.5 Da) were used to calculate relative daunomycin concentration.

### *In vivo* antitumor and anti-metastasis efficacy

The antitumor and anti-metastasis effect of liposome preparations were carried out in various murine breast cancer allograft models. First, we investigated primary tumor growth and metastasis incidence after subcutaneous, intravenous, and orthotopic (nipple and mammary fat pad) injections of cancer cells. In the case of subcutaneous and mammary fat pad models, 100 μL of cell suspension with a concentration of 5 × 10^6^ cells/mL was injected into the respective sites of mice. When injecting cells intramammary, 20 μL of cell suspension was used with a concentration of 2.5 × 10^7^ cells/mL. For intravenous injection, 50 μL of cell suspension with a concentration of 10^7^ cells/mL was applied into the tail vein of mice. After the establishment of our orthotopic murine allograft model by injecting cells into the mammary fat pads of mice, liposome formulations were tested. Once the tumor volume reached 50 mm^3^, mice were randomized and assigned to different groups for each treatment, respectively 0.9% saline as control, free daunomycin, Lipo-NP, Lipo-25S, Lipo-50S, Lipo-100S, E-Lipo-25S, Lipo-25C, Lipo-50C, and Lipo-100C. Treatments were injected intraperitoneally two times per week, five times in total (daunomycin dosage of 5 mg/kg in case of liposome formulations and 2.5 mg/kg was used for free drug – MTD). The weight and tumor size of mice were monitored during the whole experiment three times per week. On Day 27, mice were euthanized, and primary tumor, heart, lung, liver, and spleen were harvested and stored in 4% formalin (Molar Chemicals, Halásztelek, Hungary). After 2–3 days of incubation in formalin, organs were analyzed macroscopically and embedded into paraffin for hematoxylin & eosin (H&E) staining and immunohistochemistry. During the macroscopic analysis, metastatic nodules were counted under stereomicroscope on the surface of the entire lung of mice. Afterward, sections were cut and baked onto microscope slides. Since proliferation is a key factor in tumor progression, the commonly used nuclear antigen Ki-67 level was estimated. Anti-mouse polyclonal rabbit Ki-67 antibody (#15580) was obtained from Abcam, Cambridge, United Kingdom, and its corresponding rabbit polyclonal HRP-conjugated secondary antibody was obtained from BioCore Medical, Elkridge, Maryland, United States. Slides were scanned using Panoramic 250 Flesh III (3DHistech, Budapest, Hungary) slide scanner. Pictures were analyzed using the ImageJ deconvolution plug-in. The ratio of Ki-67+ cells and Ki-67- cells in tissue samples was calculated. In the case of macrometastasis analysis, all visible macro-metastatic lesions on the lung of animals from the control and treated groups were counted using a stereo microscope (Kruss MSZ5600, Kruss Optronic, Hamburg, Germany) under 7- to 45-fold magnification.

### Survival study

A survival study was performed using the same experimental parameters as used for antitumor and anti-metastatic efficacy. In contrast, mice were not euthanized on Day 27 but were kept under thorough monitoring. Mice were euthanized when we experienced paleness of the paws and muzzle, lethargy, or hypothermia; moreover, animals reached cutoff values defined before the experiment − 20% weight loss or tumor size reaching 2000 mm^3^.

### Statistical analysis

*In vitro* data was shown in mean ± standard deviation (SD), and *in vivo* data was presented in mean ± standard error of mean (SEM). Comparisons between control and treatment groups were performed by Mann–Whitney test, and a *p* value <.05 was considered to be a statistical difference (*) between groups.

For the survival experiment, the Mantel-Cox test was performed to compare the survival curves of different groups, and a *p* value <.05 was considered to be a statistical difference between groups.

Identification of outliers was performed using the interquartile range (IQR) method.

## Results

### Synthesis of the homing peptide derivatives

The synthesis of CREKA and SREKA homing peptides was carried out by SPPS using Fmoc/*^t^*Bu strategy on Fmoc-Rink-amide-MBHA resin as solid support. In the final step, isopropylidene-protected aminooxyacetic acid (> =Aoa-OH) was attached to the N-terminus of the SREKA peptide for the development of oxime linkage (Supplementary Scheme S1). In the case of the aminooxyacetyl-functionalized SREKA, the peptide derivative cleaved from the resin was purified by RP-HPLC followed by the removal of the isopropylidene protection (Aoa-SREKA-NH_2_). The salts and side products were separated by RP-HPLC before the conjugation of the functionalized peptide to the fatty acid derivative. As a control, a Cys containing homing peptide (H-CREKA-NH_2_) was also investigated for the coupling to the maleimide-functionalized DSPE-PEG phospholipid analog. The peptides were characterized by analytical RP-HPLC and mass spectrometry (Supplementary Figures S1 and S2).

**Figure 2.**
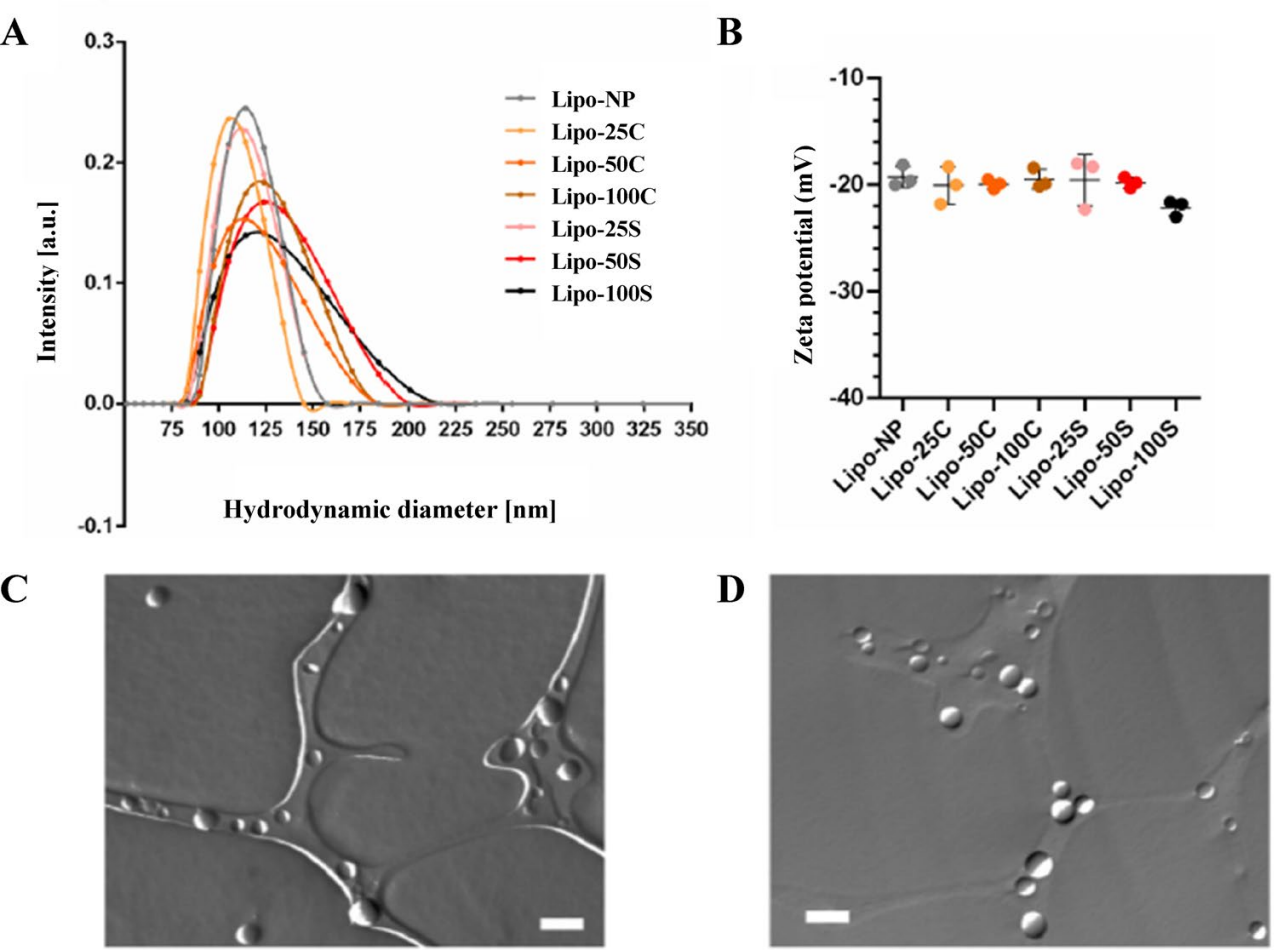
Characterization of liposome samples. Intensity weighted size distributions were measured by DLS (A) and Zeta potential values of the liposomes with different amounts of DSPE-PEG-CREKA and DSPE-PEG-SREKA (B). Representative TEM images of the Lipo-NP (C) and Lipo-100C (D) samples. Scale bars represent 200 nm.

### Conjugation of the homing peptides with the PEGylated phospholipids

The purified peptide derivatives were attached to the respective functionalized PEGylated phospholipid derivatives in two different ways to establish a stable procedure with a high yield of conjugates used for the development of targeted liposomes.

#### Method A

The aminooxyacetyl-peptide derivative was conjugated to PEGylated phospholipid (1,2-distearoyl-sn-glycero-3-phosphoethanolamine) derivative functionalized as an aldehyde (DSPE-PEG_2000_-CHO). Oxime linkage was formed under an overnight reaction between these two components (Figure 1(B); Supplementary Scheme S2(A)). The functionalized phospholipid derivatives have polydisperse molar weights; thus, the molecular set of conjugates contains several products with different molecular masses. Since the difference is due to various polymerization degrees of PEG, we can identify each derivate by looking at the length of the PEG chains. Therefore, our end-product was isolated from the reaction mixture by RP-HPLC and identified using ESI-MS (Supplementary Figure S3).

**Figure 3.**
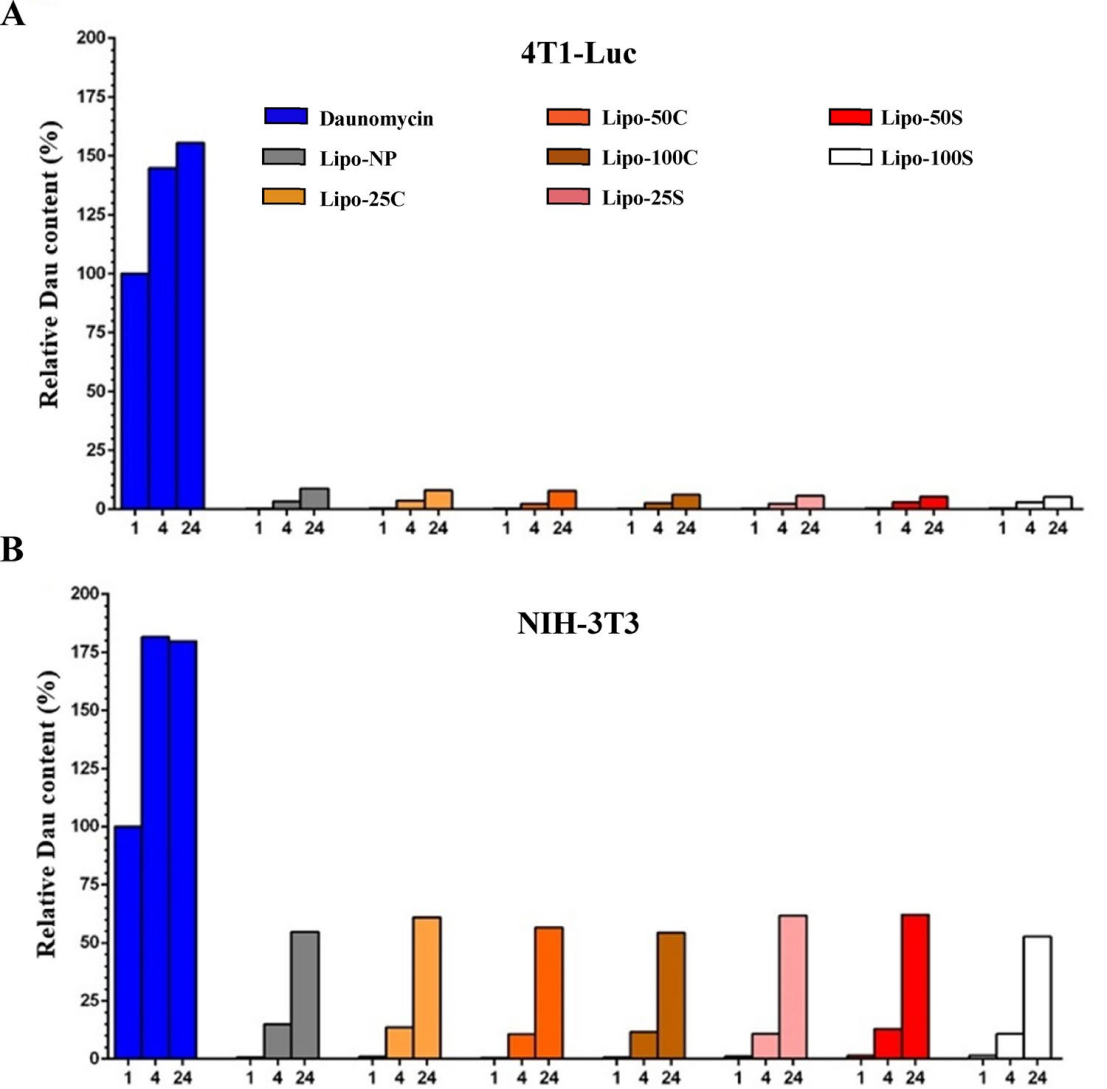
In vitro evaluation of liposome formulations and free drug using 4T1-Luc and NIH-3T3 cell lines. A and B: Cellular uptake of drug in either free from or encapsulated in liposomes. Cells were treated for 1, 4, and 24 h respectively, with an equal amount of drug either using the free drug or different liposome formulations. Drug uptake over time of 4T1-Luc (A) and NIH-3T3 (B) cell lines was measured by HPLC-MS/MS. Technical replicates (TR) = 1, biological replicate = 1.

#### Method B

The free thiol group of cysteine can attack the double bond in the maleimide ring. This well-known addition-type reaction permits conjugation between the cysteine-containing homing peptide and the DSPE-PEG_2000_-Mal derivative (Figure 1(B); Supplementary Scheme S2(B)). In this research, we followed this strategy for the development of CREKA-targeted liposomes as described previously (Zhang et al., 2017), and the end-product was obtained the same way as DSPE-PEG_2000_-SREKA conjugate (Supplementary Figure S4).

**Figure 4.**
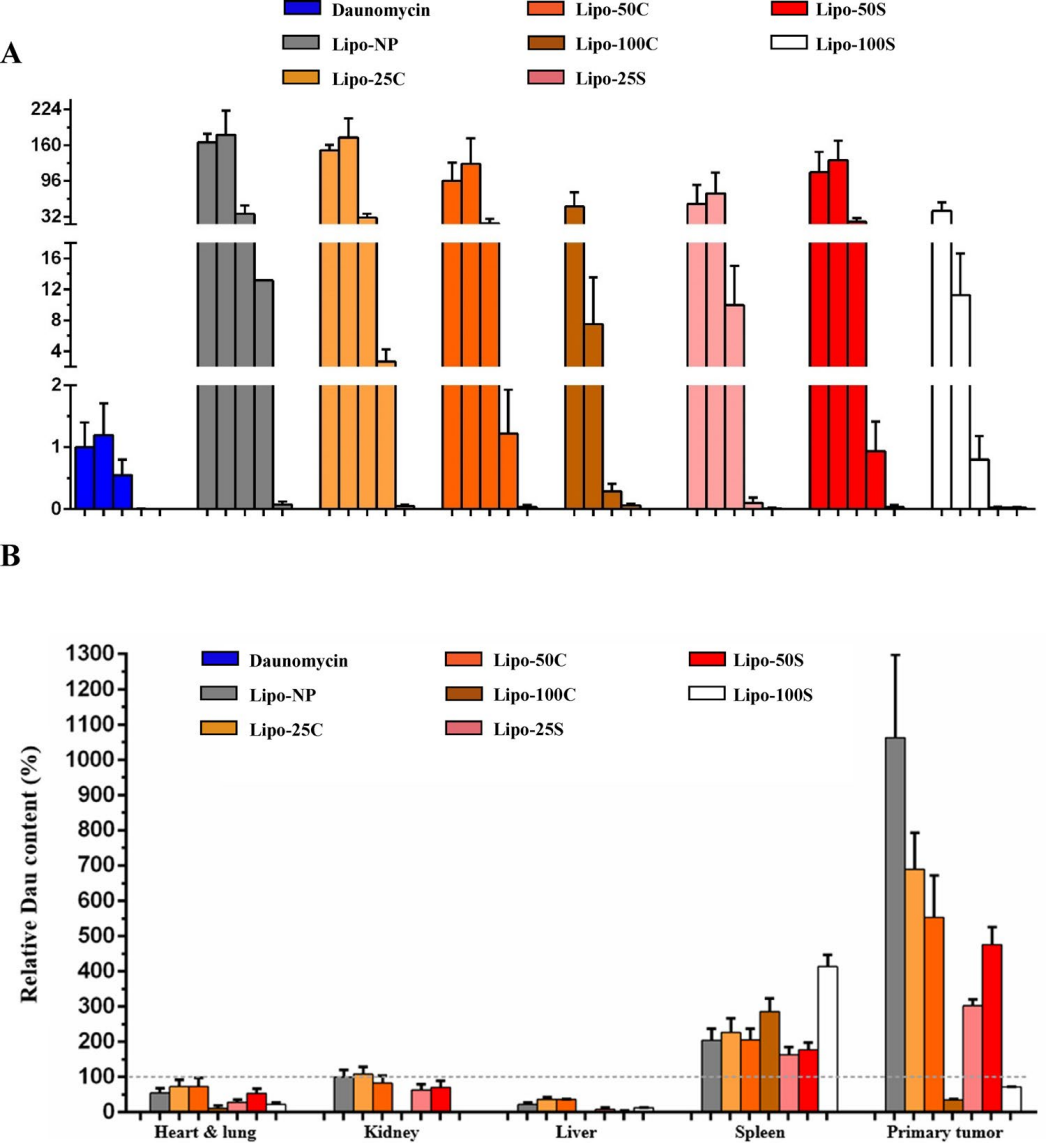
Pharmacokinetics and biodistribution of the free and liposome-encapsulated drug in murine allograft breast cancer model. Mice were treated with free drug and liposome formulations once. A: Whole blood was collected 1, 4, 24, 48, and 96 h after treatment. Total daunomycin in blood samples was extracted and measured by HPLC-MS/MS. B: Organs and primary tumors were collected from each mouse 96 h after treatment. Tissues were homogenized, and total daunomycin was extracted and detected using HPLC-MS/MS. Error bars represent the mean ± SEM. The level of daunomycin in the kidney was chosen to be 100% as it was the median value (TR = 3, BR = 3–5).

Both syntheses, *Method A* and *Method B* were repeated five times, and the yields were compared.

### Preparation and characterization of liposome formulations

The liposomal formulations used in this study resemble the liposomal formulations of daunomycin reported previously (Forssen et al., 1996; Dicko et al., 2010). Daunomycin was loaded into the liposomes with the well-known transmembrane ammonium sulfate gradient method (Haran et al., 1993; Xiong et al., 2011). Since the introduction of PEG molecules gave rise to liposomal drugs by increasing their half-life *in vivo*, attaching targeting moieties to PEGs in an extremely high amount might alter liposomal integrity. To better understand the nature of CREKA and SREKA peptides, we decided to investigate liposome preparations that were produced by adding different amounts of peptide-linked PEGylated phospholipid molecules upon liposome formulation. The terminology and their composition are shown in Table 1 (columns 1–3). ***Figure 2***(A) shows the intensity weighted size distributions of the prepared liposomes measured by DLS, and the mean diameter and polydispersity index (PDI) values are summarized in Table 1 (columns 4–5). As can be seen from these results, all samples exhibit a monodisperse distribution. However, the high amount of DSPE-PEG-CREKA (Lipo-100C) and DSPE-PEG-SREKA (Lipo-50S and Lipo-100S) shift the mean diameter toward larger values accompanied by a widening of the size distributions. This data is consistent with previous reports (Jiang et al., 2018); however, we also provided evidence that PDI values escalate with the use of an increased amount of peptide-linked PEGylated phospholipid molecules in liposome preparations, especially in the case of Lipo-100 formulations, where PDI values exceed 20%, which may result in reduced *in vivo* efficacy. Zeta potential values of the liposomal formulations are shown in Figure 2(B). All samples exhibit a Zeta potential value around –20 to –23 mV, which corresponds to good colloidal stability considering the additional steric stabilization due to the presence of PEG on the liposomal surface. Representative TEM images of the Lipo-NP (no targeting peptide) and Lipo-100C samples are shown in Figures 2(C,D), respectively. Liposomes with regular spherical shape in the 100 nm size range can be observed in the images indicating ideal liposomal morphology. TEM images of all formulations can be found in Supplementary Figure S5. Daunomycin concentration of all samples determined by UV–vis spectroscopy is summarized in Table 1 (column 6). When calculating the entrapment efficiency (EE%), we observed that it did not depend on the amount of targeting moiety used for the liposome preparations. High EE% was obtained for all formulations, ranging from 89 to 101%. The drug-to-lipid ratio (*D*/*L* ratio) was between 0.48 to 0.54, equivalent to a drug loading percentage (DL%) of 22.5 to 24.9% if the cholesterol content is not considered.

**Figure 5.**
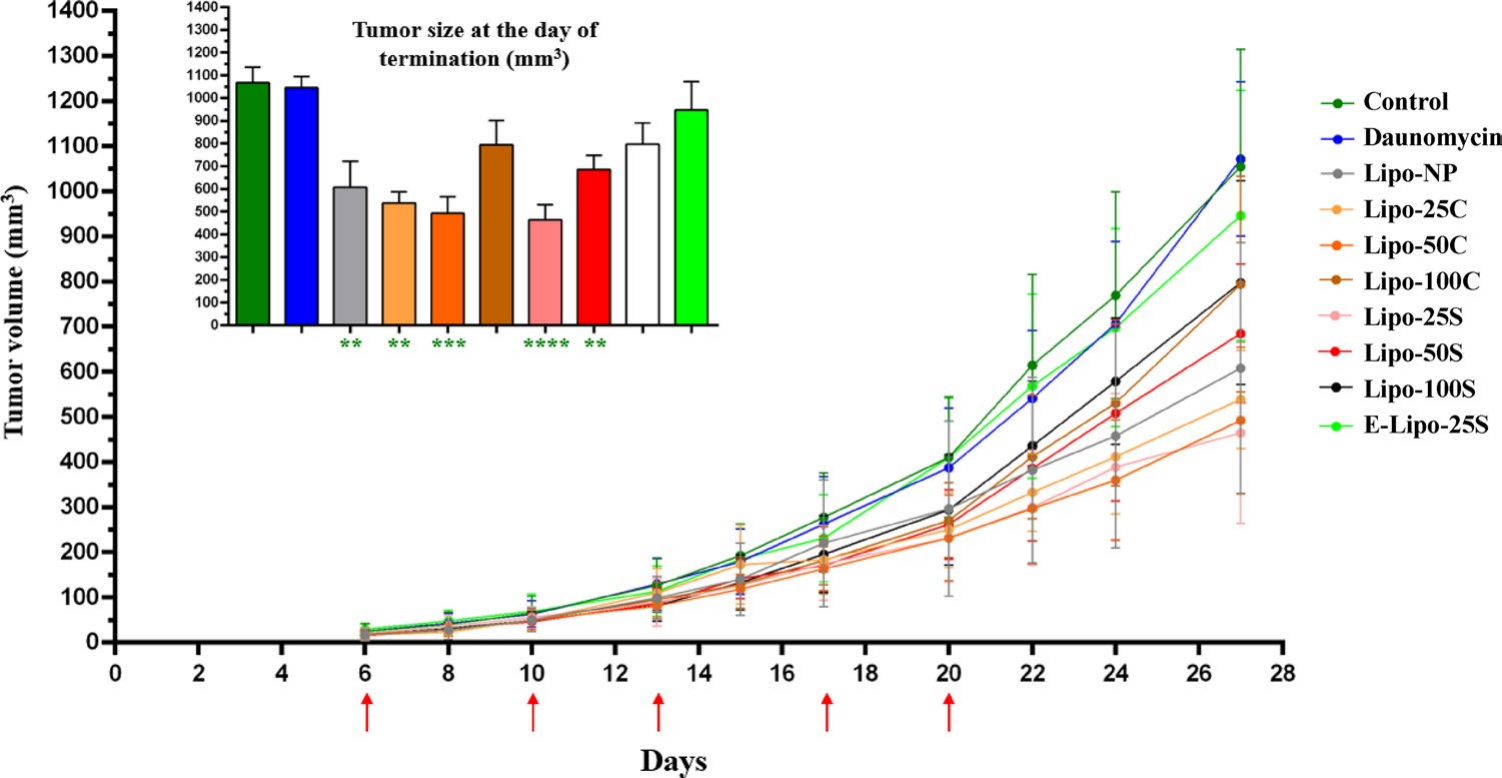
Inhibition of primary tumor expansion. Change of primary tumor size of mice treated with liposome formulations and free drug. Tumor volume is represented in mm^3^. *SL = Significance level. Error bars represent mean ± SEM (BR > 5, ***p < .001).

### *In vitro* evaluation of liposomes

To evaluate the cytotoxicity of the liposomes compared to the free drug, we performed proliferation inhibition assays with different treatment time points using a 4T1-Luc triple-negative murine breast cancer cell line (***Table 2***, Supplementary Figure S6). According to previous study (Randelovic et al., 2019) and our results, the free drug inhibits proliferation already after a 24-h treatment, and it does not have a stronger effect in the case of longer treatment. Conversely, liposome preparations had a stronger effect after a 72-h continuous treatment, resulting in lower IC_50_ values compared to 24-h treatment. IC_50_ values decreased 2.3-times with Lipo-NP, 7.2-times with Lipo-50S, and 4.8-times with Lipo-100S, respectively.

**Figure 6.**
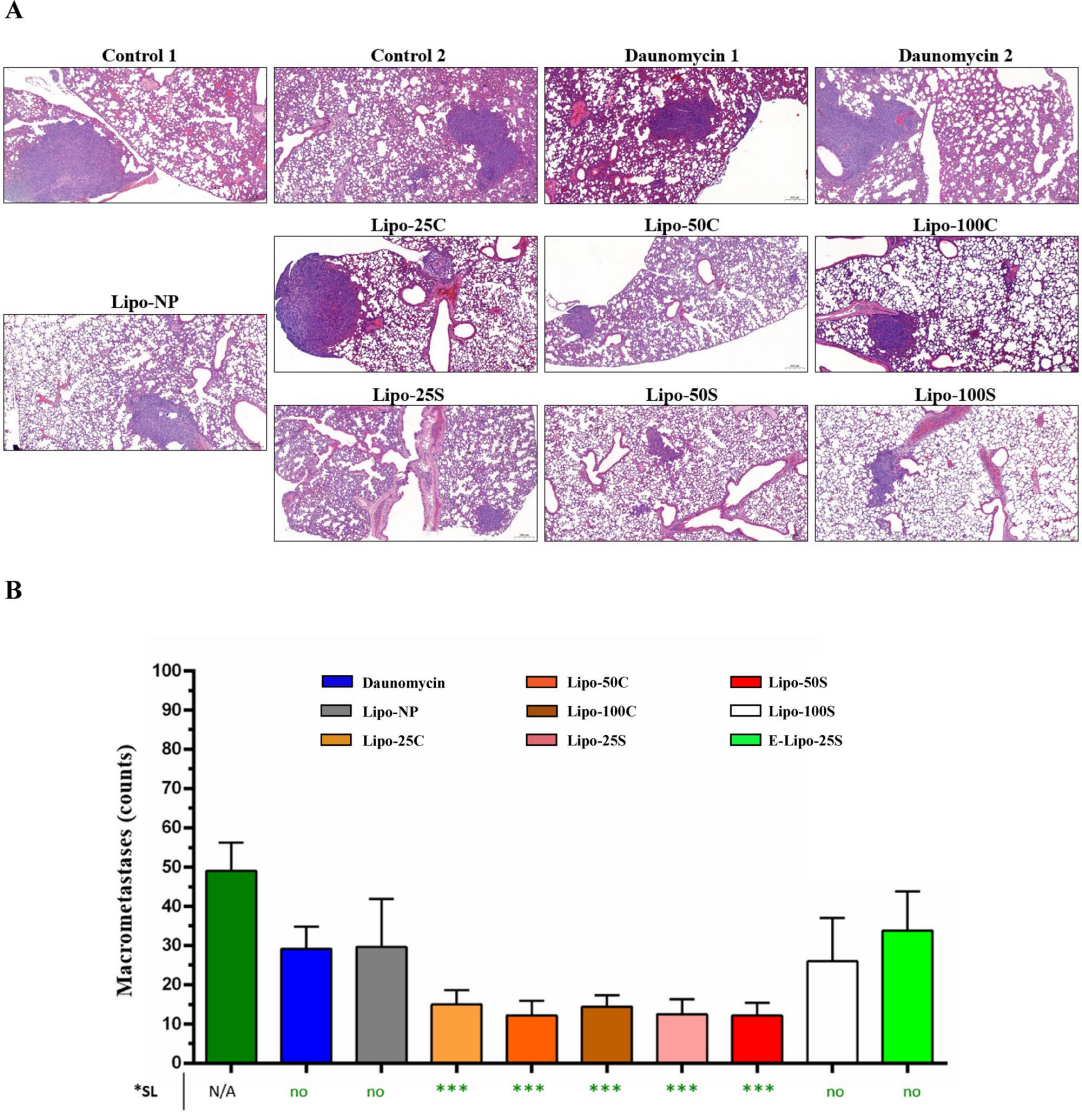
Inhibition of metastatic incidences in the lung. A: H&E staining of the lung of mice treated with liposome formulations and free drug. B: The number of metastatic nodules on the surface of the entire lung of mice treated with mock treatment, free daunomycin and liposomal formulations. *SL = Significance level. Error bars represent the mean ± SEM (*n* > 5, ***p < .001).

**Table 2.** In vitro antiproliferative evaluation of liposome formulations and free drug using 4T1-Luc cells.

	IC50 value – nM (72 h)	IC50 value – nM (24 + 48 h)
Daunomycin	90.4 ± 1.14	88.95 ± 1.3
Lipo-NP	163 ± 1.25	379 ± 1.3
Lipo-50S	79.4 ± 1.24	572.5 ± 1.37
Lipo-100S	119.5 ± 1.28	573.9 ± 1.29

Cells were treated for 24 h, then free drug and liposome preparations were exchanged to growth medium. After 48 h of incubation, living cells were detected. Cells were treated continuously for 72 hours, then living cells were detected. Data are represented as the average of three experiments ± SD. Technical replicates (TR) = 3, biological replicates (BR) = 3.

**Table 3.** Biodistribution of the free and liposome-encapsulated drug in murine allograft breast cancer model.

	Daunomycin	Lipo-NP	Lipo-25C	Lipo-50C	Lipo-100C	Lipo-C avg	Lipo-25S	Lipo-50S	Lipo-100S	Lipo-S avg
Heart & Lung	N/A	43.4%	56.1%	44.21%	0%	33.4%	24.8%	45.6%	25.6%	32%
Kidney	N/A	100%	110%	71.3%	0%	60.4%	58.1%	58.2%	0%	38.8%
Liver	N/A	24.7%	41.9%	61.3%	0%	34.4%	0%	0%	23.7%	7.9%
Spleen	N/A	232.2%	247.1%	243.5%	359.7%	283.3%	207.6%	230.3%	591.6%	343.2%
Primary tumor	N/A	1006.3%	825.8%	533.4%	59%	472.7%	452.9%	643%	123.3%	406.4%

Mice were treated with 3 mg/kg free daunomycin, or with liposome formulations containing equal amount of drug. Organs were harvested 96 h after treatment and total daunomycin was measured using HPLC. (TR = 3, BR = 3-5).

To better understand this phenomenon, cellular uptake of the free drug was compared to the uptake of the drug delivered by liposome preparations using the 4T1-Luc cell line (***Figure 3***(A)). Since free daunomycin is transported into cells via passive diffusion (Siegfried et al., 1985; Willingham et al., 1986), its level was already high 1 h after treatment, and it continued to increase until 4 h after treatment. On the contrary, uptake of daunomycin was prolonged in the case of liposome formulations, resulting in a low level of the drug 1 h after the treatment and a continuously increasing amount over time. These results are consistent with the outcome of our proliferation inhibition studies suggesting that liposomes extend and stabilize the release of their cargo. Since fibroblast is a type of cell that contributes to the formation of the connective tissues by secreting fibrous cellular material, we expect to see higher uptake of liposomes by these cells (Brissett & Hom, 2003). To examine whether a higher amount of target molecules results in faster uptake of the drug, we measured daunomycin uptake in NIH-3T3 murine fibroblasts (Figure 3(B)). As hypothesized, NIH-3T3 cells showed an elevated level of daunomycin uptake compared to 4T1-Luc cells, indicating that a higher level of fibronectin and fibrin capture the liposomes more efficiently and promote drug release.

### Liposome stability in whole blood and biodistribution

To investigate the behavior of liposomes *in vivo*, various murine experimental models were established by implanting 4T1-Luc cells into Balb/c mice (Supplementary Figure S7). Cells were injected subcutaneously, intravenously, into the nipple and mammary fat pad of mice. Based on survival, tumor growth, and metastasis incidence (data not shown), we decided to proceed with the experimental model when cells were injected into the mammary fat pad of mice.

**Figure 7.**
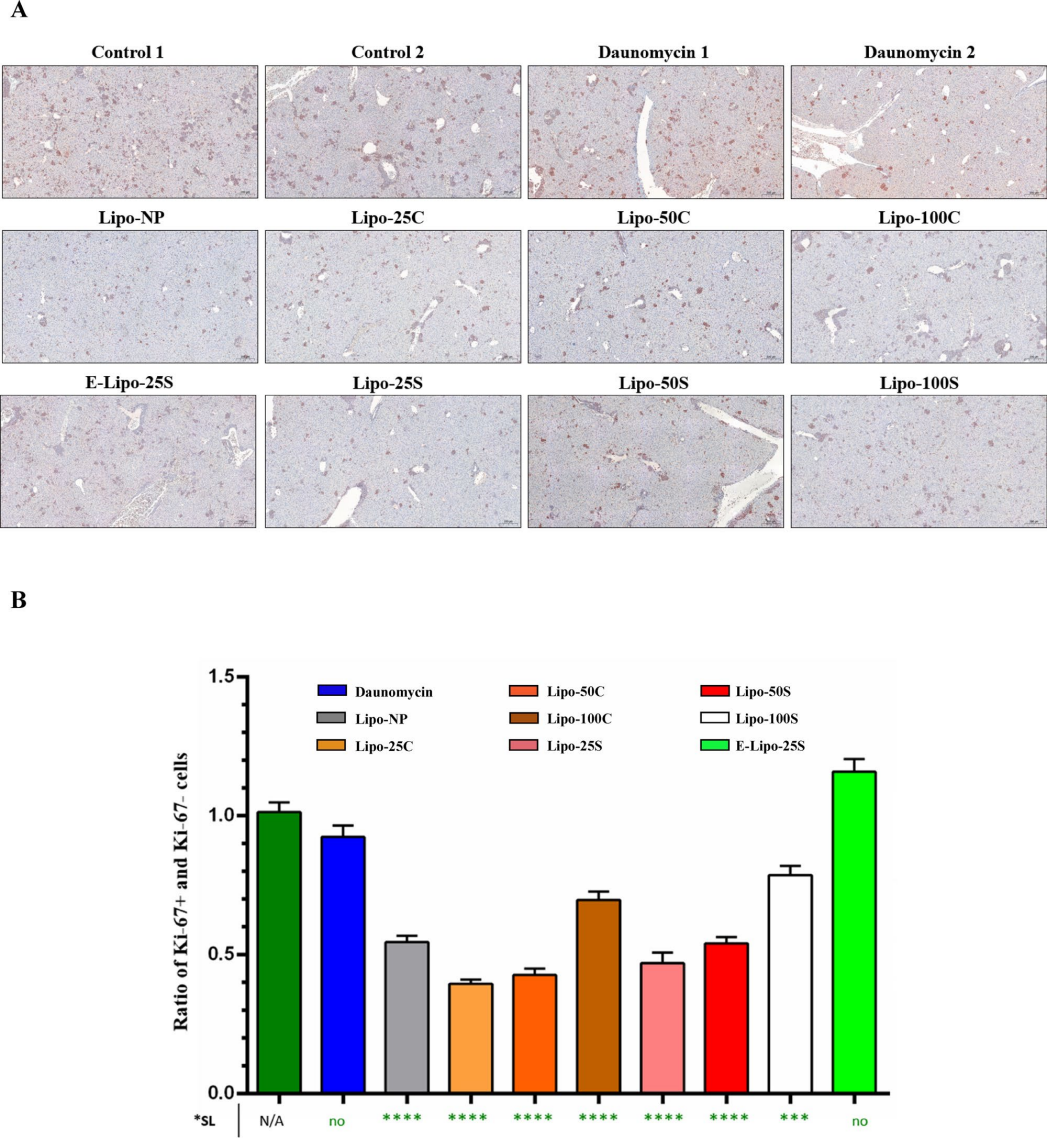
Inhibition of metastatic incidences in the liver. A: Ki-67 staining of the liver obtained from mice treated with free daunomycin and liposomal formulations. B: The ratio of metastatic and healthy cells in the liver of mice from different treatment groups. *SL = Significance level. Error bars represent the mean ± SEM (*n* = 15, ***p < .001).

Mice were treated with an equal amount of daunomycin (3 mg/kg) either by using the free drug or liposome formulations, and the level of the drug was determined in whole blood using HPLC-MS/MS method (***Figure 4***(A)). Our results are consistent with other studies showing that drug encapsulated in liposomes has a longer half-life compared to the free drug (Wang et al., 2013; Li et al., 2015; Jiang et al., 2018). Moreover, we also showed that Lipo-100S and Lipo-100C seem to be less efficient in maintaining the level of daunomycin in the bloodstream compared to the other liposome formulations, as drug concentration is substantially lower already at 24 h after treatment.

Afterward, we evaluated which organs are most affected upon treatment with free daunomycin and liposome preparations. Ninety-six hours after treatment, primary tumor and major organs such as lung, heart, liver, kidney, and spleen were removed from mice. After homogenization of tissues, HPLC-MS/MS measurement was performed to detect daunomycin in each organ (Figure 4(B) and Table 3). According to our results, drug content was undetectable in all tissue types when treating mice with the free drug. This may be due to the previously mentioned fast clearance of free daunomycin (Wang et al., 2013; Li et al., 2015; Jiang et al., 2018), which was completely cleared from the bloodstream at 96 h after treatment. In the case of most liposome preparations, we can see the accumulation of the drug in the analyzed tissues. Lipo-C formulations delivered a relatively higher amount of daunomycin into the heart, lung, kidney, and liver compared to Lipo-S formulations. The mean drug content released from the different Lipo-C preparations into the heart and lung, kidney, and liver were 1.05, 1.6, and 4.4 times higher than in the case of Lipo-S, respectively. On the other hand, Lipo-S formulations seemed to deposit more drug into the spleen on average; however, it might be due to the fact that the treatment with Lipo-100S resulted in extremely high accumulation compared to all other liposome formulations. Lipo-25S and Lipo-50S showed lower drug levels in the spleen compared to their Lipo-C counterparts.

Finally, we showed that Lipo-NP deposits the highest amount of daunomycin in the primary tumor compared to other liposome formulations. In addition, we can observe that Lipo-25 and Lipo-50 formulations also delivered the highest amount of daunomycin into the primary tumor when compared to other tissues; however, these liposome preparations did not reach the efficiency of Lipo-NP.

### Antitumor and antimetastatic efficacy of liposomes

Antitumor efficacy of liposome formulations was next tested *in vivo* using our allograft experimental model (***Figure 5***(A)). Although the free drug was used at a maximum tolerated dose (Supplementary Figure S8), it was not able to slow down primary tumor growth compared to the control (*p*_daunomycin_ = .5765). Moreover, treatment with E-Lipo-25S (not daunomycin-loaded) neither resulted in a smaller tumor size, and interestingly, Lipo-100C and Lipo-100S were also unable to significantly decrease tumor development compared to the control (*p*_Lipo-100C_ = .0606; *p*_Lipo-100S_ = .0516). This data supports our hypothesis that the stability of Lipo-100C and Lipo-100S is lower compared to other liposome formulations, which results in less efficient targeting. In contrast, Lipo-NP inhibited the increase in primary tumor size, but its antitumor efficacy was still lower than Lipo-25S, Lipo-25C, and Lipo-50C formulations. Lipo-50S also reduced tumor growth compared to the control, but Lipo-NP treatment resulted in a somewhat lower primary tumor size (*p*_Lipo-NP_ = .002; *p*_Lipo-25C_ = .0013; *p*_Lipo-50C_ = .0006; *p*_Lipo-25S_ < .0001; *p*_Lipo-50S_ = .0032). The body weight of mice did not change considerably throughout the experiment (Supplementary Figure S9) in the case of groups treated with liposome formulations; however, Lipo-50C, Lipo-100C, Lipo-100S, and Lipo-NP treatment decreased the mean body weight by over 5%. Altogether, this data suggests great safety and low toxicity of liposomes.

**Figure 8.**
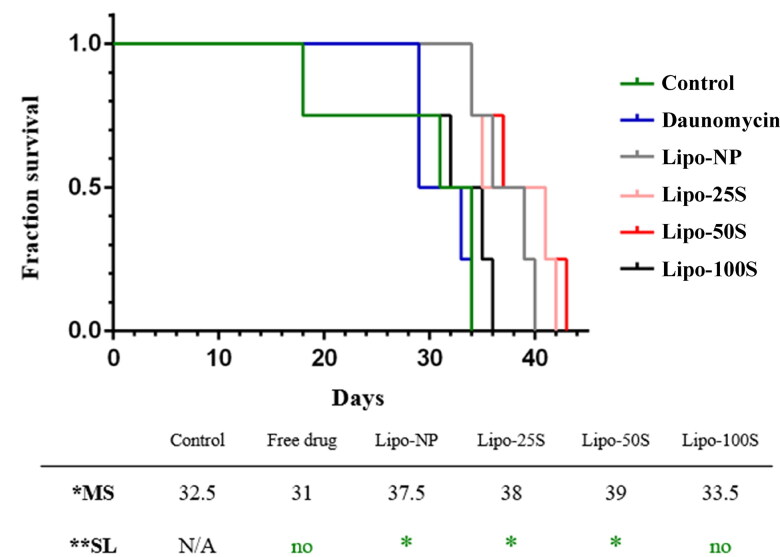
Survival of murine allograft breast cancer model upon treatment with the free and liposome-encapsulated drug. The survival of mice (from the time tumor cells were injected until reaching cutoff values) is compared by the Mantel-Cox test between controls and groups treated with either free drug or drug encapsulated in liposomes. The median survival of different groups is shown in the table below. *MS = Median survival in days. **SL = Significance level. (*n* > 4, *p < .05).

Since most breast cancer-related deaths are due to metastases at distant organs (Weigelt et al., 2005), next, we examined the antimetastatic effect of liposome preparations at two common metastatic sites of breast cancer, lung, and liver, respectively. Lipo-25S, Lipo-50S, Lipo-25C, and Lipo-50C significantly inhibited the formation of metastatic sites in the lung compared to the control (***Figure 6***(B)). Moreover, Lipo-100C was also able to interrupt metastases, contrary to free daunomycin, E-Lipo-25S, Lipo-100S, and Lipo-NP (*p*_daunomycin_ = .818; *p*_Lipo-NP_ = .1721; *p*_Lipo-25C_ = .0003; *p*_Lipo-50C_ = .0003; *p*_Lipo-100C_ = .0003; *p*_Lipo-25S_ =.0001; *p*_Lipo-50S_ = .0001; *p*_Lipo-100S_ = .099). These observations were confirmed by H&E staining of lung sections derived from the animals (Figure 6(A)). Proliferation is one of the key markers in tumor progression; therefore, immunohistochemical staining was performed to identify cells expressing nuclear antigen Ki-67 in the liver of mice (***Figure 7***(A)). Since healthy liver cells do not express high levels of Ki-67 (King et al., 1998), an increased level of expression might indicate aggressive and rapidly progressing tumors. Besides the positive Ki-67 signal, these cells exhibited an irregular shape which was not characteristic of the Ki-67- healthy cells. We calculated the ratio of Ki-67+ cells and Ki-67– cells to investigate whether or not liposome preparations were able to inhibit tumor progression into the liver (Figure 7(B)). Our results indicate that all liposome formulations significantly reduced the number of Ki-67+ cells compared to the control, except for E-Lipo-25S (*p*_daunomycin_ = .1679; *p*_Lipo-NP_ < .0001; *p*_Lipo-25C_ < .0001; *p*_Lipo-50C_ < .0001; *p*_Lipo-100C_ < .0001; *p*_Lipo-25S_ <.0001; *p*_Lipo-50S_ < .0001; *p*_Lipo-100S_ = .0002). However, the mean ratio of Ki-67+ and Ki-67– cells was only lower in Lipo-25C, Lipo-50C, Lipo-25S, and Lipo-50S compared to Lipo-NP.

### Survival of mice upon liposome treatment

We also assessed the survival of tumor-bearing mice upon treatment with SREKA-directed liposomes (***Figure 8***). Survival curves indicate that free daunomycin treatment did not expand lifespan compared to the control, and neither did Lipo-100S. On the contrary, Lipo-25S and Lipo-50S significantly increased life expectancy resulting in 16%–20% growth in lifespan compared to the control (*p*_daunomycin_ = .6696; *p*_Lipo-NP_ = .0285; *p*_Lipo-25S_ = .0285; *p*_Lipo-50S_ = .0285; *p*_Lipo-100S_ = .3076). In addition, although, Lipo-NP did not affect metastasis incidence in the lung, besides a significantly smaller primary tumor size and a significant inhibition of cancer expansion to the liver, mice treated with Lipo-NP had a longer lifespan as well compared to the controls.

## Discussion

The use of liposomes as drug delivery systems is a relatively new field that started with Doxil® in 1995 (Barenholz, 2012). Since then, numerous nanodrugs have entered the clinical practice, and several others are being tested in clinical trials (Ventola, 2017). Although the application of liposomal drugs still has its challenges, including the necessity of better characterization, understanding how the integrity of liposomes remains intact upon the employment of targeting moieties, and the development of cost-effective production. However, previous studies show exceptionally promising results with CREKA-targeted liposomes on highly aggressive triple-negative breast cancer models (Wang et al., 2015; Zhang et al., 2017; Jiang et al., 2018), but clinical use is still not applicable. There might be several reasons for this, such as unstable chemical reactions while synthesizing CREKA, inefficient conjugation of DSPE-PEG to CREKA, high costs for liposome production, or low stability of liposomes when prepared with CREKA-modified PEGylated phospholipids. In this study, we focused on two major challenges mentioned earlier: cost-effectivity and the integrity of liposomes when decorating them with targeting moieties.

Since cysteines are extremely vulnerable against oxidation, the conjugation could be a challenging approach. Even though, serious efforts were made to optimize the reactions with cysteines, there is still room for improvement in cost effectivity. In our opinion, it might be a better approach not to optimize the experiment itself, but to exchange the vulnerable amino acid to a more stable one with similar characteristics. When the oxidability of cysteine is addressed in chemical experiments, in most cases cysteine is replaced with alanine or serine. In spite of serine being more hydrophilic than cysteine, our choice was based on the geometric similarity that is usually crucial for target binding. Since SREKA resulted in similar characteristics and activity compared to CREKA, with the exchange of cysteine for serine, we aimed to develop a more cost-effective approach for the conjugation of the targeting moieties to DSPE-PEG molecules. From a starting mass of 20 mg functionalized DSPE-PEG derivatives, the average yields after HPLC purification were 4.3 and 3.2 mg conjugates for SREKA and CREKA peptides, respectively. During CREKA conjugation, a high amount of disulfide dimer formation could be observed, which might be the explanation for the substantial decrease in the yield compared to SREKA. The higher amount (ca. 34%) of oxime-linked conjugate compensates for the higher price (ca. 25%) of the aldehyde functionalized DSPE-PEG over DSPE-PEG-Mal, which results in more cost-effective production of liposomal material. This significantly lower the costs of liposome production; however, the experiment can still be optimized further for better cost-effectivity. When comparing the liposomes in functional experiments, we did not see significant differences between CREKA and SREKA as targeting moieties.

Recent evidence suggests that cellular uptake of the free drug is much more efficient *in vitro* compared to its liposomal form (Zaleskis et al., 2021). Here, we confirmed this phenomenon, when monitoring uptake and antiproliferative abilities of free drug and liposomal formulation; however, we also demonstrated that after 72 h of treatment, liposomes have the same antiproliferative effect as the free drug *in vitro*. Moreover, we showed that a higher level of fibronectin excreted in culture flasks results in increased cellular uptake of liposomes. This indicates that liposome uptake is more specific than passive diffusion. Additionally, encapsulation does not decrease the potency of free drug, but it prolongs cellular uptake *in vitro*.

Numerous liposomal formulations are used in clinics and are under clinical investigation; however, the effect of introducing targeting moieties is still subject to controversy. PEG molecules are responsible for the increase of liposomal half-life by inhibiting phagocytosis (Blume & Cevc, 1990). Since targeting moieties are attached to PEG, the stability of liposomes could be altered upon this modification. Lower stability results in premature drug release *in vivo*; therefore, targeting efficiency of liposomes may be moderate, resulting in higher toxicity and worse selectivity toward cancer-associated tissue.

Here, we showed that the mean diameter of liposome formulations containing a high amount of targeting peptide is shifted to the larger values, and their size distribution is widening compared to other liposome preparations. However, we could not identify significant differences among the liposome formulations in our *in vitro* proliferation inhibition assay.

Intraperitoneally administered PEGylated liposomes go through the lymphatic system to reach the bloodstream (Allen et al., 1993). Since the spleen is part of the lymphatic system and acts as one of the first barriers for liposomes, we expected to see a higher accumulation of daunomycin in case of treatment with unstable liposomes. According to our results, Lipo-100 formulations deposit higher levels of the drug into the spleen. Moreover, daunomycin level detected in blood also decreases significantly in the case of Lipo-100 formulations already 24 h after treatment compared to Lipo-25 and Lipo-50 formulations. When investigating daunomycin levels in the primary tumor, we showed that Lipo-NP had the highest amount compared to the other formulations. This may be due to Lipo-NP’s great stability and its lack of targeting metastatic sites. Since CREKA and SREKA target fibronectin and fibrin that are most abundant in metastatic sites, we hypothesize that Lipo-S and Lipo-C formulations deposit their cargo primarily at metastatic sites instead of the primary tumor site. Furthermore, we reported that Lipo-25 and Lipo-50 formulations deposit more drug to the primary tumor than to any other tissue, showing that liposomes with targeting moiety keep their affinity toward primary tumor tissue as well.

It is also worth mentioning that Lipo-25S and Lipo-50S did not deposit detectable levels of daunomycin into the liver. Besides clearance by the kidney, daunomycin would be metabolized in the liver; therefore, this may be an indication of lower toxicity of Lipo-S formulations compared to Lipo-C formulations.

Taken together, higher accumulation of daunomycin in the spleen, lower level of drug in the tumor tissue, and fast clearance from blood indicate premature drug release due to lower liposome stability. Therefore, this supports our hypothesis that the integrity of Lipo-100 formulations is lower compared to Lipo-25 and Lipo-50 formulations, which result in worse targeting ability and higher toxicity, even though Lipo-100 liposomes have a higher amount of targeting moiety. Synthesizing high amounts of targeting moieties and conjugating them to the delivery systems even for *in vivo* studies using murine models, but especially for clinical studies require a vast amount of time and funding. Our data elucidates that sometimes a little goes a long way, meaning that applying lower amount of targeting moieties which is just enough to have affinity toward the target site would be beneficial over decorating liposomal formulations with high amounts of targeting molecules.

Considering the effect on tumor expansion and survival, we demonstrated that Lipo-25 and Lipo-50 formulations show significant inhibition of primary tumor growth, as well as prevention of metastasis progression. Moreover, mice treated with Lipo-25S and Lipo-50S had a significantly higher lifespan compared to controls. Since Lipo-NP is based on Doxil®, which is currently used in clinics (Barenholz, 2012), it is not surprising that we observed great antitumor efficacy and better survival upon treatment compared to control. However, However, it has been shown previously that CREKA-targeted liposomes outmatch their non-targeted counterparts (Jiang et al., 2018). Here, we confirmed these results, moreover, we reported that the more cost-effective Lipo-25S and Lipo-50S formulations also outperformed Lipo-NP. This data indicates that the effect of Lipo-25 and Lipo-50 formulations are more than passive targeting on the tumor tissue.

Further work is needed to investigate the applicability of SREKA-targeted liposomes for clinical use. First, the conjugation of SREKA to DSPE-PEG molecules can be further optimized to reach an even better yield. Another possible advancement is the encapsulation of more potent chemotherapeutic agents. Daunomycin is a well-known intercalating agent used in clinics in free and liposomal forms. Nowadays, patients are not treated with the free drug, but it has great potential for screening targeting molecules. Since we aimed to have better comparability to the literature, we also used daunomycin as the active agent. On the other hand, liposomal formulations have the benefit that various chemotherapeutic agents can be loaded into them. Therefore, inhibition of tumor expansion potency of SREKA-targeted liposomes, when loaded with more potent, experimental model-specific drugs, should be further investigated.

To conclude, in this study, we illustrated a well-established method to stably produce our newly developed SREKA pentapeptide, conjugate it to DSPE-PEG molecules in a scalable way, and produce liposomes using different ratios of modified and non-modified PEGylated phospholipid molecules. We showed that despite a higher amount of targeting moiety would suggest higher specificity and better targeting, Lipo-100 formulations performed poorly in the inhibition of tumor expansion, as well as in increasing the survival of tumor-bearing mice. Moreover, we showed that this new method for the development of peptide-targeted liposomes using Ser instead of Cys is more cost-effective and can be used to prepare various types of targeted liposomes with different peptide moieties.

## Supplementary Material

Supplemental Material

## Data Availability

All data generated or analyzed during this study are included in this published article [and its supplementary information files]. Moreover, rare datasets are available on the repository: zenodo.org upon request, under the DOI: 10.5281/zenodo.7075436.
