# Supplementary material for: SREKA-targeted liposomes for highly metastatic breast cancer therapy
Source: Drug Deliv. 2023 Feb 8;30(1):2174210. doi: 10.1080/10717544.2023.2174210 (PMC9930758; doi:10.1080/10717544.2023.2174210)
Supplement: Supplemental Material [file IDRD_A_2174210_SM8999.docx]

**Scheme S1**: Synthesis of the *Aoa*-SREKA-*NH_2_* peptide derivative, as a representative example for the synthesis of homing peptides

A)





B)





**Scheme S2**: Synthesis of the DSPE-PEG_2000_-CH_2_-C=N-O-CH_2_-CO-SREKA-*NH_2_* (**A**) and DSPE-PEG_2000_-MAL-S-(*H*-CREKA-*NH_2_*) conjugate (**B**)

Analytical chromatograms of compounds:

Analytical RP-HPLC was performed on a Waters Symmetry (WAT 045905) C18 column (150x4.6mm I.D.) with 5 µm silica (100 Å pore size) as a stationary phase. A linear gradient elution was developed: 0 min 0% B; 2 min 0% B; 22 min 90% B with eluent A (0.1% TFA in water) and eluent B (0.1% TFA in acetonitrile-water (80: 20, v/v)). A flow rate of 1 mL/min was used at ambient temperature. Samples were applied and dissolved in eluent A and 20 μl was injected. Peaks were detected at λ = 220 nm.


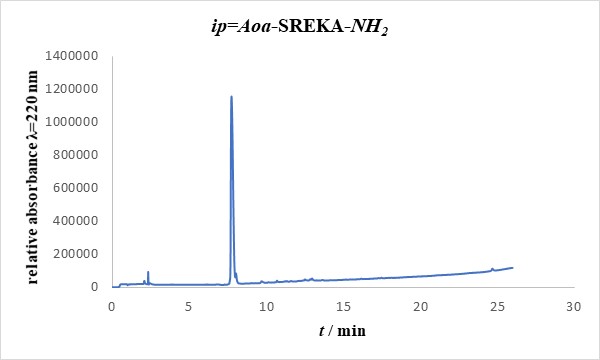

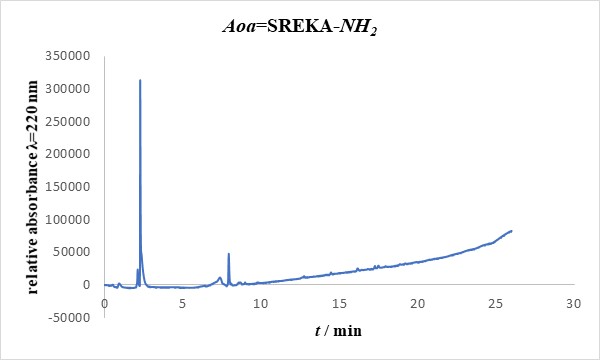

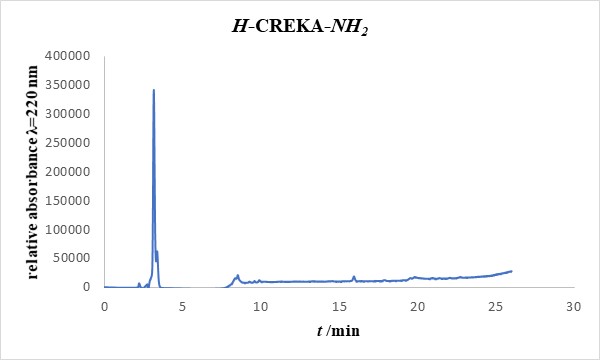


**Figure S1**: Analytical chromatogram of the peptide derivatives

Mass spectrometry

The peptide analogs and conjugates were identified by electrospray ionization mass spectrometry (ESI-MS) on a Bruker Daltonics Esquire 3000 Plus (Bremen, Germany) ion trap mass spectrometer, operating in continuous sample injection at 4 µL/min flow rate. Samples were dissolved in ACN-water (50:50 v/v%) mixture containing 0.1 v/v% AcOH. Mass spectra were recorded in positive ion mode in the *m/z* 50-2000 range.

A)


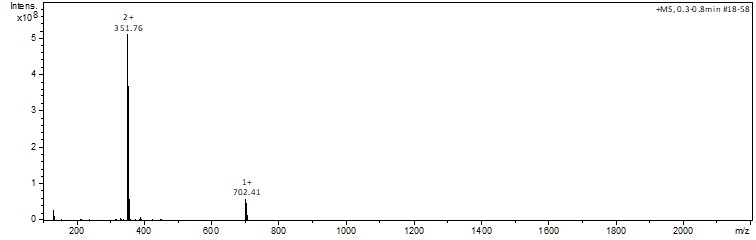


B)


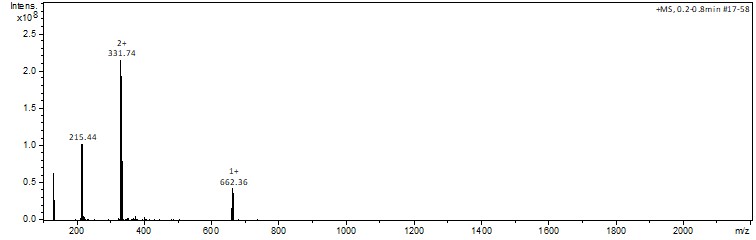


C)

**Figure S2**: Mass spectra of *ip=Aoa*-SREKA-*NH_2_* (A), *Aoa*-SREKA-*NH_2_* (B), and *H*-CREKA-*NH_2_* (C)


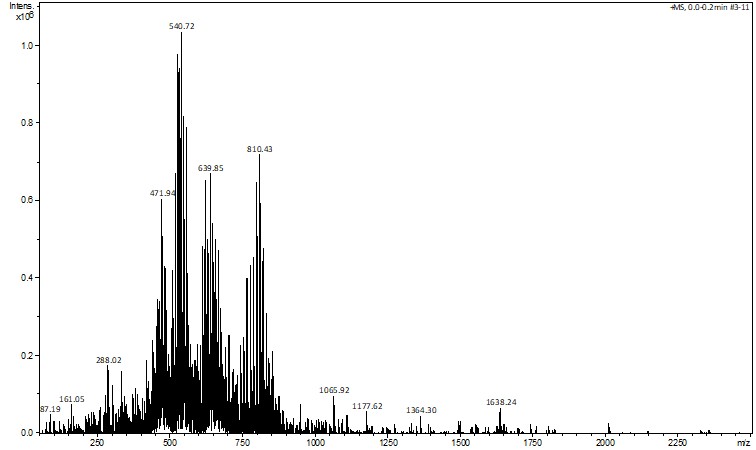


44.0262
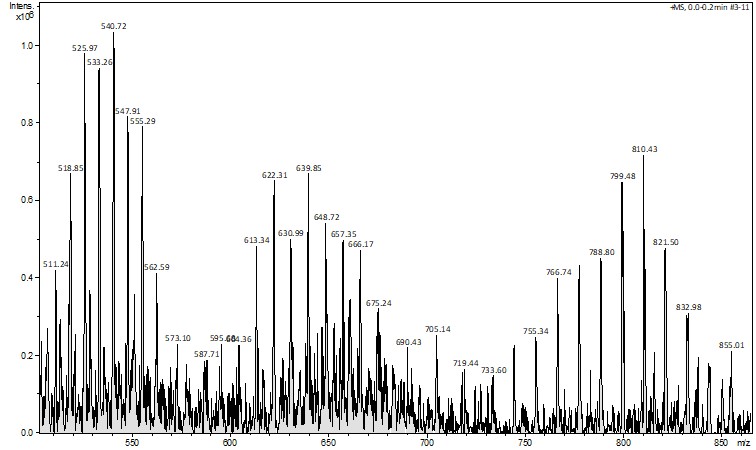


M_Wcalc_(DSPE-(CH_2_-CH-O)_n_-(CH_2_-CH=N-O-CH_2_-CO)-SREKA-*NH_2_*

**1520.944585 (n=1)**

**[(n-1) x 44.0262 +** **1520.944585 Da (n=1)]**

**Figure S3**: Mass spectra of the DSPE-PEG(2000)-CH_2_-CH=N-O-CH_2_-CO-SREKA-*NH_2_*


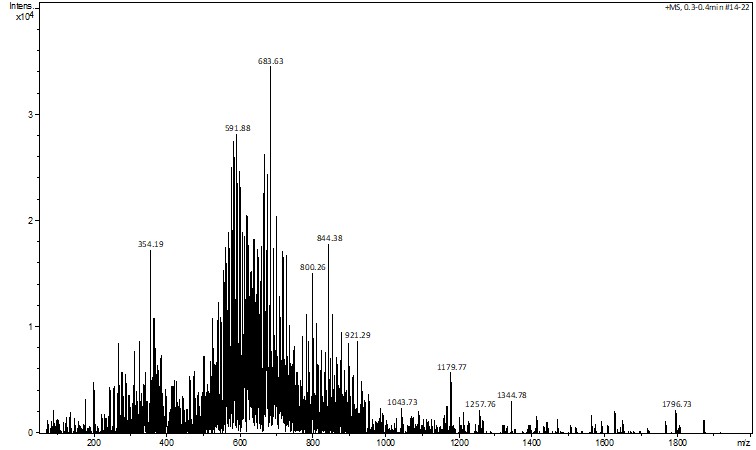


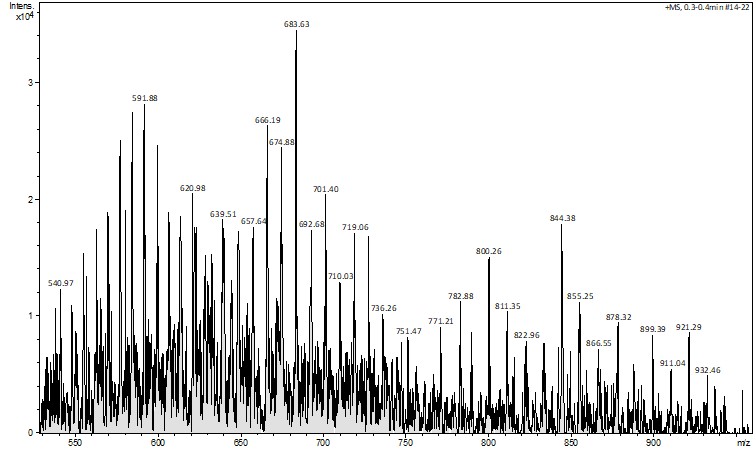


M_Wcalc_(DSPE-(CH_2_-CH-O)_n_-)-(CH_2_)_2_-NH-CO-(CH_2_)_2_-O)-MAL-*H*-CREKA-*NH_2_*

**Mw: 1633.9634 (n=1)**

**[(n-1) x 44.0262 +** **1633.9634 + 22 (Na+) Da]**

**Figure S4**: Mass spectra of the DSPE-PEG(2000)-(CH_2_)_2_-NH-CO-(CH_2_)_2_-O)-MAL-*H*-CREKA-*NH_2_*


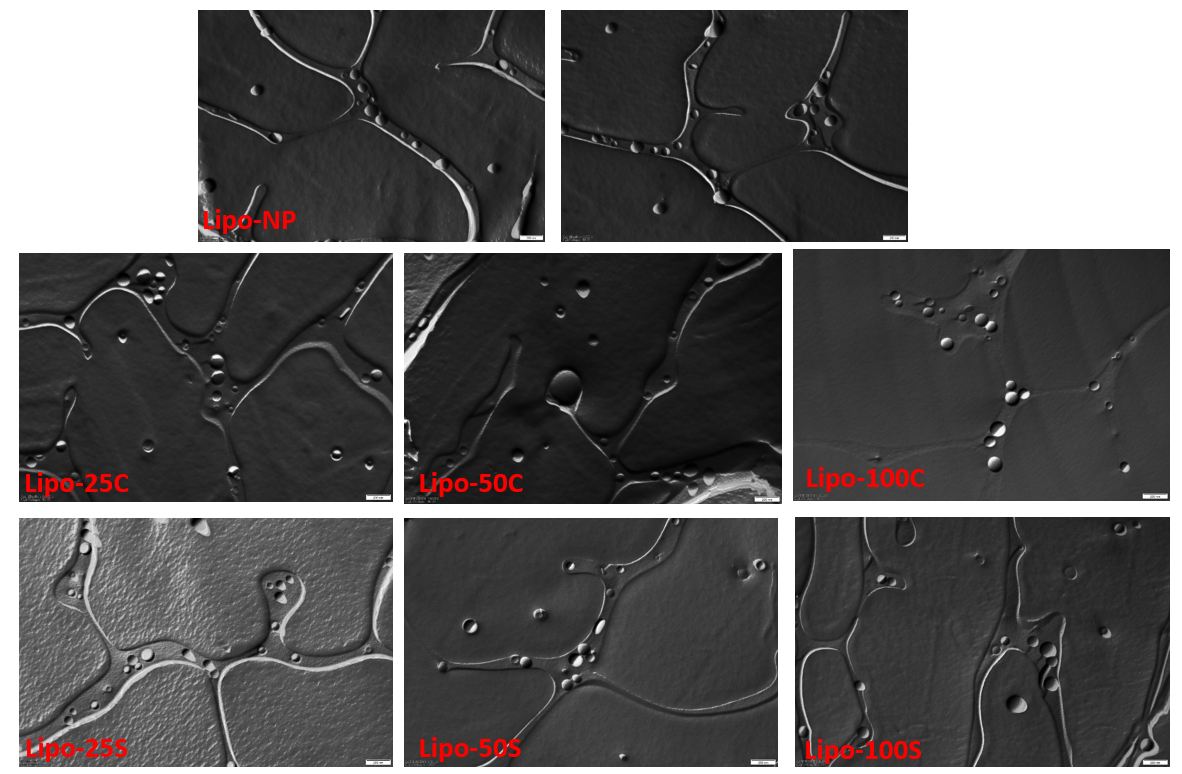


**Figure S5: FF-TEM pictures of all liposome preparations**. Scale bars represent 200 nm.


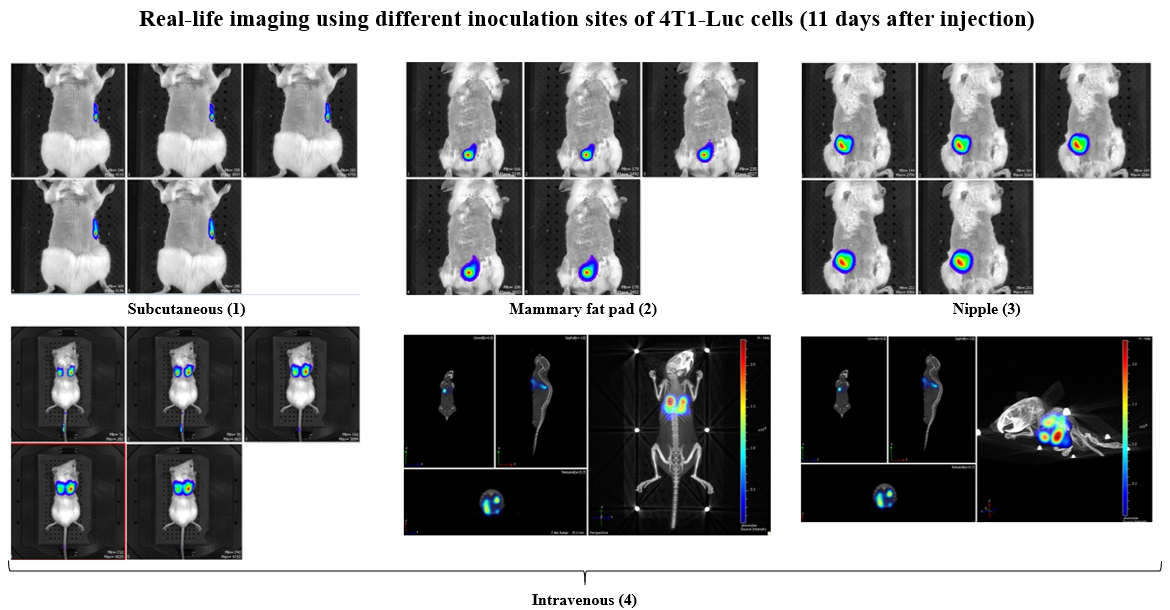


**Figure S6: Real-life imaging of 4T1-Luc-bearing Balb/c mice**. Cells were injected in four different ways, subcutaneously (1), into the mammary fat pad (2), into the nipple (3), or intravenously (4), respectively. The bioluminescent signal is derived from the activity of 4T1-Luc cells upon intraperitoneal injection of luciferin into mice. Models 1, 2, and 3 show that injected cells adhere to the injected tissue and establish a solid tumor at the injection site. Model 2 shows that cells are transferred into the chest of mice and colonize the lung.

**
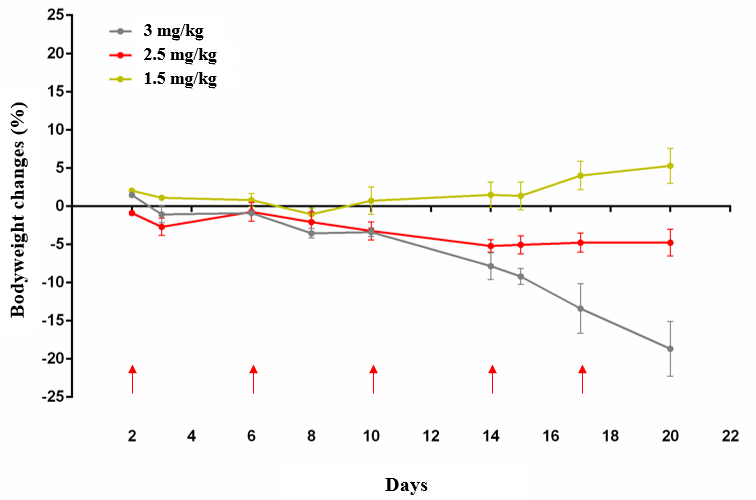
Figure S7: Toxicity of free Daunomycin on Balb/c mice.** Mice were treated with free Daunomycin five times in total (indicated with red arrows) in different concentrations (grey line 🡪 3 mg/kg, red line 🡪 2.5 mg/kg, yellow line 🡪 1.5 mg/kg). Each group contained 3 mice. The mean of bodyweight change is indicated. Error bars represent the mean of bodyweight changes ± SEM.


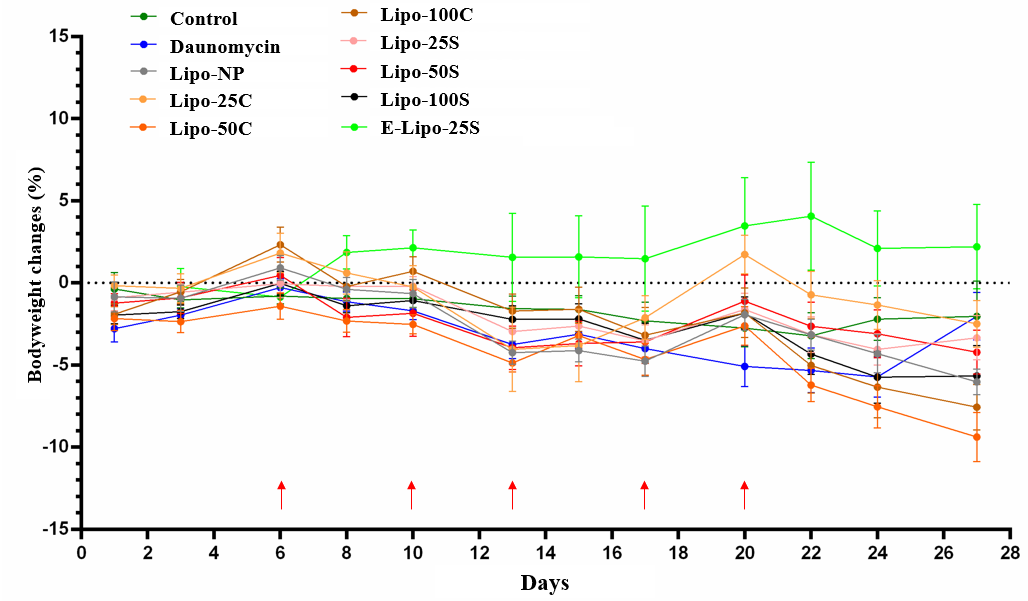


**Figure S8: Change of bodyweight of mice upon treatment with mock treatment, free drug, and liposome formulations.** Red arrows indicate treatment time points. Each color represents the group labeled on top of the graph. Bodyweight was calculated the following way: (“actual weight” – “starting weight”) / “starting weight x 100”. Error bars represent the mean of bodyweight changes ± SEM.
